# A guide through the computational analysis of isotope-labeled mass spectrometry-based quantitative proteomics data: an application study

**DOI:** 10.1186/1477-5956-9-30

**Published:** 2011-06-11

**Authors:** Stefan P Albaum, Hannes Hahne, Andreas Otto, Ute Haußmann, Dörte Becher, Ansgar Poetsch, Alexander Goesmann, Tim W Nattkemper

**Affiliations:** 1 Computational Genomics, Center for Biotechnology (CeBiTec), Bielefeld University, Germany; 2 Biodata Mining Group, Faculty of Technology, Bielefeld University, Germany; 3 Bioinformatics Resource Facility, CeBiTec, Bielefeld University, Germany; 4 Chair for Proteomics and Bioanalytics, Center of Life and Food Sciences Weihenstephan, Technische Universität München, Germany; 5 Institute of Microbiology, Ernst-Moritz-Arndt-University Greifswald, Germany; 6 Plant Biochemistry, Ruhr University Bochum, Germany; 7 Current Address: Chair for Proteomics and Bioanalytics, Center of Life and Food Sciences Weihenstephan, Technische Universität München, Germany

## Abstract

**Background:**

Mass spectrometry-based proteomics has reached a stage where it is possible to comprehensively analyze the whole proteome of a cell in one experiment. Here, the employment of stable isotopes has become a standard technique to yield relative abundance values of proteins. In recent times, more and more experiments are conducted that depict not only a static image of the up- or down-regulated proteins at a distinct time point but instead compare developmental stages of an organism or varying experimental conditions.

**Results:**

Although the scientific questions behind these experiments are of course manifold, there are, nevertheless, two questions that commonly arise: 1) which proteins are differentially regulated regarding the selected experimental conditions, and 2) are there groups of proteins that show similar abundance ratios, indicating that they have a similar turnover? We give advice on how these two questions can be answered and comprehensively compare a variety of commonly applied computational methods and their outcomes.

**Conclusions:**

This work provides guidance through the jungle of computational methods to analyze mass spectrometry-based isotope-labeled datasets and recommends an effective and easy-to-use evaluation strategy. We demonstrate our approach with three recently published datasets on *Bacillus subtilis *[1,2] and *Corynebacterium glutamicum *[3]. Special focus is placed on the application and validation of cluster analysis methods. All applied methods were implemented within the rich internet application QuPE [4]. Results can be found at http://qupe.cebitec.uni-bielefeld.de.

## Background

Developments in the field of mass spectrometry over the last decade have brought the analysis of proteins to a new level, and allow today's scientists to comprehensively scrutinize these integral components of life that act as molecular machines, structural elements, transporters, or receptors [5]. In high-throughput experiments, liquid chromatography coupled to tandem mass spectrometry (LC-MS/MS) is utilized to characterize the complete set of proteins contained in a cell or organism. Recent methods, moreover, employ isotopic labels to enable the quantification of proteins [6-14]. Datasets resulting from such quantitative proteomics experiments are often very complex and consist of lists of measured abundance values for hundreds (or thousands) of proteins. As a manual exploration of such large datasets is practically impossible, there is a strong need for computational approaches concerning statistical data analysis and data mining in order to support experimenters. The scientific questions being addressed by these experiments are without any doubt very different. There are, however, two questions that commonly arise: 1) which proteins are differentially regulated regarding the selected experimental conditions, and 2) are there groups of proteins that are characterized by similar abundance ratios, indicating a common regulation? Aim of this work is to answer these two questions considering-as application example-three real-world datasets on *Bacillus subtilis *[1,2] and *Corynebacterium glutamicum *[3], thereby taking into account the particular challenges of mass spectrometry-based proteomics data.

### Question 1)

Obviously, each time a measurement of a protein's abundance is performed, an-albeit small-variation in the recorded value may occur. This variation may have different origins, and that is what needs to be determined: are the changes governed by regulatory mechanisms in a cell, e. g. as a response to a stress stimulus an organism is exposed to, or do they originate from other sources such as a natural fluctuation or technical errors in the process of measurement itself. Given a number of measured abundance ratios for a protein, a small variation between these values could mean that the strict enforcement of the protein's quantity is of key importance, e. g. for the development of an organism. Contrary, a rather high variation could indicate a weak influence of regulatory elements and lead to the assumption that the exact dosage e. g. of an enzyme regarding a metabolic pathway may not be important. If, for a protein, repeated measurements are obtained under different conditions, i. e. can be separated into two or more groups, it can be questioned whether variations are larger between two groups than within the same group. In order to assess the significance of deviations a statistical test such as the analysis of variance (ANOVA) may be employed. A meaningful interpretation of the results, however, demands certain prerequisites: i) within a group, deviations from the group's mean value should follow a Gaussian-distribution, ii) the samples should be taken from equally distributed populations; therefore, variances within different samples are not allowed to differ significantly, and iii) the influence of confounding variables has to be independent for each measurement. Infringements of these premises, in particular of ii, might result in the false assessment of proteins as significantly differentially regulated. Although the ANOVA has more power in terms of discovering significant differences, in cases of violated assumptions a non-parametric method such as the Kruskal-Wallis one-way analysis of variance has to be applied [15,16].

### Question 2)

In the analysis of these complex datasets one is often interested in the determination of protein groups that show similar changes in abundance in relation to the experimental conditions. It seems reasonable to suppose that these proteins are commonly regulated or functionally related. A computational method to identify groups of proteins with similar abundance profiles is cluster analysis. Belonging to the group of unsupervised learning methods, cluster analysis is characterized in that no external information is needed. The operation is solely performed on inherent features of the data-clusters are not known *a priori *but discovered during the clustering process. The aim of clustering is to aggregate a number of measurements, i. e. proteins, in groups, so called clusters, such that all members of a group are as homogeneous as possible, while at the same time requiring that there is a considerable heterogeneity between all elements of two clusters [17,18]. Clustering techniques are traditionally divided into three distinct classes: a) hierarchical, b) partitioning or vector quantization, and c) probabilistic or density-based methods [19,20]. a) (Agglomerative) hierarchical approaches group objects into clusters, which in turn are iteratively grouped into clusters, thereby forming a hierarchical tree structure [21-23]. b) Following a given optimization strategy and a specified number of groups partitioning approaches assign each individual to one distinct group. One of the most prominent algorithms is K-means [24,25]. c) Density-based approaches differ from the other two strategies in the way that each object not necessarily belongs to a single cluster but instead is assigned a probability that specifies its membership to a group. An example is fuzzy C-means clustering [26].

Cluster analysis has the potential to reveal hidden structures in the data, which-in the context of quantitative proteomics-might be groups of proteins having a similar pattern of regulation. However, the validity of the outcome of an unsupervised learning method such as cluster analysis is difficult to assess (cf. i.a. [18]). In the run-up to the analysis, in general, no information regarding a true clustering is available. Moreover, the results produced by different algorithms are (very) often dissimilar: the hierarchical structures for example obtained by Single- and Complete-linkage are seldom characterized by a strong congruence. A fundamental part of the clustering process therefore is an evaluation of the algorithm's results [27,28], which, to our knowledge, has been discussed for other "omics"-data but so far not for quantitative proteomics datasets.

## Results and Discussion

Our study is based on three real-world datasets. Two experiments on *Bacillus subtilis *consist of each three biological replicate measurements, and describe a time series of five distinct time points. In experiment A, samples were taken directly after a salt stress was induced and after 10, 30, 60, and 120 minutes [1]. In experiment B, which unveils temporal changes in the proteome caused by glucose starvation, cells were harvested during exponential growth, and 0, 30, 60, and 120 minutes after transition from exponential to stationary growth phase [2]. A third experiment C investigates the adaption of *Corynebacterium glutamicum *to alternative carbon sources [3]. In contrast to the aforementioned experiments, two different growth media-benzoate and glucose-were examined. It was, moreover, decided to include only one replicate in this analysis to demonstrate the applicability of the provided evaluation strategy on smaller datasets. Please note therefore that the following analysis results of this experiment are not comparable to the results presented in the original, very comprehensive proteomics study. The two questions to answer in all three experiments are: 1) which proteins are differentially regulated regarding the factor time (A, B) or, in case of experiment C, regarding the factor carbon source, and 2) are there groups of proteins that show a similar pattern of regulation in terms of their relative abundance.

For experiment A and B, Mascot (TM) [29] was used for protein identification, for experiment C existing identifications resulting from Sequest (TM) [30] were imported in QuPE [4]. After quantification using QuPE's built-in algorithm, in experiment A abundance ratios had been calculated for 58,895 peptides leading to 1,285 different quantified proteins with at least one measurement for at least one time point; in experiment B for 180,913 peptides amounting to 2,321 proteins, and in experiment C for 3,699 peptides and 589 proteins.

### Question 1) Detection of differentially regulated proteins

An approach commonly applied to detect differentially regulated proteins is based on the determination of a user-defined threshold in form of a x-fold change in abundance. This method, however, has one significant drawback as it inevitably ignores the different types of variability of a sample. Instead, it is important to find out whether replicate measurements belonging to a protein show a larger variability between different conditions than within the same group [31]. This requires statistical analysis methods such as the one-way analysis of variance (ANOVA). Prior to the application, the highest acceptable significance level *α *has to be set-common values are 0.05 or 0.01. Considering a single statistical test one may allow an error of as much as *α *to falsely reject the null hypothesis. Albeit small for a single test, this error increases dramatically when multiple tests have been performed. This is certainly the case in quantitative proteomics experiments where hundreds to thousands of proteins are investigated in a single experiment. Therefore, this "family-wise error rate" (FWER), which defines the probability that at least one of this type I errors might occur, should be taken into consideration [32-34]. To account for the multiple testing situation all computed *p*-values should be corrected using a method such as proposed by Holm [33]. As already mentioned above, the ANOVA demands certain prerequisites to be fulfilled: i) the assumption that all residues, i. e. deviations from the group's mean, follow a normal distribution can be investigated using a Shapiro-Wilks test [35]; ii) to analyze the homogeneity of variances of each group a Fligner-Killeen test may be utilized [36]. In order to circumvent these requirements, the non-parametric Kruskal-Wallis rank sum test (KW) may be employed as alternative to an ANOVA.

In the present work, we want to determine if both methods detect the same proteins as significantly differentially regulated. In view of the limited number of biological replicates for all three experiments, statistical tests were performed on every peptide measurement, i. e. each abundance ratio determined by a ^15^N-labeled/unlabeled peptide pair was considered as an independent measurement of the protein's quantity. If **x **= {*x_i_*, *i *= 1, . . . , *N*} is a series of calculated relative abundance values for a specific protein, and **t **denotes an equally-sized vector which assigns each value *x_i _*a fixed time point *t_i_*, the (fixed effects) model for the two experiment A and B can be defined as follows:(1)

In the third experiment C, instead of time the factor carbon source **c **applies, and each value *x_i _*is assigned either the condition benzoate *c*_1 _or glucose *c*_2_. This leads to the following model:(2)

#### Evaluation of statistical tests

The acceptable significance level *α *was set to 0.05, and all computed *p*-values where corrected by Holm's method. For experiment A, the ANOVA revealed 73 proteins being significantly differentially regulated regarding the five time points (see Table 1 and Additional file 1). However, the Fligner-Killeen test (ii) indicated that 15 of these proteins have inhomogeneous variances. Using the Shapiro-Wilks test (i), moreover, in 29 cases the normal distribution assumption was violated. Taking this into account, strictly speaking, only 38 proteins can therefore be regarded as significantly differentially regulated. The Kruskal-Wallis rank sum test found 64 proteins with significant change in their abundance (see Table 2). In comparison, from the 38 proteins that fulfilled the strict requirements of the ANOVA, 21 were not found significantly regulated by the Kruskal-Wallis test. However, ignoring the strict requirements of normally distributed residues and homogeneous variances, more than 80% of the proteins (52) that were declared significant by the ANOVA were likewise assessed by the Kruskal-Wallis test.

**Table 1:** Significantly expressed proteins - ANOVA result

Accession	ANOVA adjusted p-values	Fligner-Killeen adjusted p-values	Shapiro-Wilks adjusted p-values	Kruskal-Wallis adjusted p-values	Number of identified peptide hits
**P40780**	<0.000001	>0.99	>0.99	<0.000001	741

O34833	<0.000001	0.007364	<0.000001	0.000001	827

O32076	<0.000001	0.000083	>0.99	<0.000001	1344

O34538	<0.000001	0.000020	0.977644	<0.000001	1616

**P94565**	<0.000001	>0.99	0.264667	0.000003	1033

P54466	<0.000001	0.003793	<0.000001	<0.000001	2382

P33166	<0.000001	<0.000001	0.067524	<0.000001	1605

P02968	<0.000001	0.000001	<0.000001	<0.000001	9369

P37809	<0.000001	0.000002	0.007523	<0.000001	1112

P46920	<0.000001	>0.99	<0.000001	<0.000001	1149

**P09124**	<0.000001	>0.99	>0.99	<0.000001	685

P35136	<0.000001	>0.99	0.000003	<0.000001	1498

**P37808**	<0.000001	>0.99	>0.99	<0.000001	1219

P94356	<0.000001	0.037934	0.414572	<0.000001	2053

**P80877**	<0.000001	>0.99	>0.99	0.000002	892

P27206	<0.000001	0.000004	0.000028	<0.000001	2232

**P37871**	<0.000001	0.399160	0.901907	<0.000001	1938

O34992	<0.000001	0.085647	<0.000001	<0.000001	1215

P37476	<0.000001	>0.99	<0.000001	<0.000001	3203

Q04747	<0.000001	<0.000001	0.000199	<0.000001	3460

**O31709**	<0.000001	>0.99	>0.99	0.000020	295

P35149	<0.000001	0.000169	>0.99	0.000693	751

P08065	<0.000001	0.328408	<0.000001	<0.000001	2140

P19582	<0.000001	>0.99	0.011528	0.000008	903

O34966	<0.000001	0.012613	0.000188	<0.000001	1837

P08750	<0.000001	>0.99	<0.000001	<0.000001	1109

O34442	<0.000001	>0.99	0.047638	0.000552	291

P24141	0.000001	>0.99	<0.000001	<0.000001	1257

P25994	0.000001	0.755894	0.006728	0.000072	1641

**O32243**	0.000003	0.599332	>0.99	0.000505	404

O06745	0.000005	>0.99	0.013199	0.011193	485

**P37527**	0.000013	>0.99	>0.99	*0.051621*	181

**P04969**	0.000016	>0.99	>0.99	*0.708837*	745

O32167	0.000020	>0.99	0.001374	0.000021	2891

O32157	0.000035	0.016583	<0.000001	0.000796	957

O31501	0.000036	0.006965	<0.000001	<0.000001	1681

P20166	0.000046	0.272716	0.044373	0.000003	1948

**P21471**	0.000052	>0.99	>0.99	*0.558509*	130

**P26906**	0.000063	>0.99	>0.99	*0.875231*	300

P54535	0.000073	>0.99	<0.000001	<0.000001	2391

**O31663**	0.000143	>0.99	>0.99	*0.378199*	340

**O34633**	0.000185	>0.99	>0.99	0.025892	433

**P09339**	0.000203	0.244699	>0.99	0.040871	619

**O07516**	0.000230	>0.99	>0.99	*0.955961*	370

P24327	0.000234	>0.99	<0.000001	<0.000001	1692

**P37494**	0.000244	>0.99	>0.99	*0.932235*	241

**P32399**	0.000267	>0.99	>0.99	0.045315	388

**P39912**	0.000327	>0.99	>0.99	0.002252	878

**P42971**	0.000473	>0.99	>0.99	0.032372	475

**P21472**	0.000594	>0.99	>0.99	***¿ **0.99*	357

P21464	0.000613	>0.99	<0.000001	0.000473	1272

**P71021**	0.000703	>0.99	>0.99	***¿ **0.99*	173

O31567	0.001315	>0.99	0.000289	0.002616	613

**O06491**	0.001750	>0.99	>0.99	*0.542278*	268

**O34789**	0.001860	>0.99	>0.99	***¿ **0.99*	106

**O35007**	0.002474	>0.99	>0.99	0.011411	410

P71044	0.002703	>0.99	0.000005	0.000284	635

**P12042**	0.002703	>0.99	>0.99	0.043005	699

**P46921**	0.003377	>0.99	>0.99	*0.421630*	173

**P28611**	0.004644	>0.99	>0.99	*0.193988*	259

**P13484**	0.006385	>0.99	>0.99	***¿ **0.99*	208

**P39215**	0.006851	0.484875	>0.99	*0.275825*	608

**O32218**	0.009165	>0.99	>0.99	0.018027	567

**P18185**	0.011352	>0.99	>0.99	***¿ **0.99*	220

P21467	0.015346	>0.99	0.000001	0.001040	781

P37870	0.017921	0.000748	>0.99	0.041718	1113

**O31657**	0.018006	>0.99	>0.99	*0.111378*	227

**O06478**	0.022471	>0.99	>0.99	*0.859843*	229

**O34878**	0.022750	>0.99	>0.99	***¿ **0.99*	83

**O32247**	0.025031	>0.99	>0.99	0.028009	845

**P39694**	0.031255	>0.99	0.293920	***¿ **0.99*	96

**P42319**	0.038119	>0.99	>0.99	***¿ **0.99*	224

P49785	0.039250	>0.99	0.000245	0.002557	536

Exemplary shown for experiment A, this table displays all ANOVA-reported significantly expressed proteins having a *p*-value below the acceptable significance level of *p *< 0.05 (further results can be found in the supplementary data). Proteins are sorted by the ANOVA's *p*-value in ascending order. The assumption that all values are derived from a normal distribution was investigated using a Shapiro-Wilks test: in 29 cases the normal distribution assumption was violated. To analyze the homogeneity of variances of each group a Fligner-Killeen test was performed. Here, 15 of the proteins showed inhomogeneous variances. In summary, only 38 proteins fulfilled all criteria (in bold print). Interestingly, 21 of these proteins were not found significantly regulated by a Kruskal-Wallis test (in italics). All computed *p*-values where corrected using the method described by Holm [33].

**Table 2:** Significantly expressed proteins - Kruskal-Wallis result

Accession	ANOVA adjusted p-values	Fligner-Killeen adjusted p-values	Shapiro-Wilks adjusted p-values	Kruskal-Wallis adjusted p-values	Number of identified peptide hits
**P40780**	<0.000001	>0.99	>0.99	<0.000001	741

**P24327**	0.000234	>0.99	<0.000001	<0.000001	1692

**O32076**	<0.000001	0.000083	>0.99	<0.000001	1344

**P24141**	0.000001	>0.99	<0.000001	<0.000001	1257

**O34538**	<0.000001	0.000020	0.977644	<0.000001	1616

**P54535**	0.000073	>0.99	<0.000001	<0.000001	2391

**P54466**	<0.000001	0.003793	<0.000001	<0.000001	2382

**P02968**	<0.000001	0.000001	<0.000001	<0.000001	9369

**O31501**	0.000036	0.006965	<0.000001	<0.000001	1681

**P46920**	<0.000001	>0.99	<0.000001	<0.000001	1149

**O34966**	<0.000001	0.012613	0.000188	<0.000001	1837

**P35136**	<0.000001	>0.99	0.000003	<0.000001	1498

**P37808**	<0.000001	>0.99	>0.99	<0.000001	1219

**P94356**	<0.000001	0.037934	0.414572	<0.000001	2053

**P27206**	<0.000001	0.000004	0.000028	<0.000001	2232

**P37871**	<0.000001	0.399160	0.901907	<0.000001	1938

**O34992**	<0.000001	0.085647	<0.000001	<0.000001	1215

**P37476**	<0.000001	>0.99	<0.000001	<0.000001	3203

**Q04747**	<0.000001	<0.000001	0.000199	<0.000001	3460

**P08750**	<0.000001	>0.99	<0.000001	<0.000001	1109

**P33166**	<0.000001	<0.000001	0.067524	<0.000001	1605

**P08065**	<0.000001	0.328408	<0.000001	<0.000001	2140

**P09124**	<0.000001	>0.99	>0.99	<0.000001	685

**P37809**	<0.000001	0.000002	0.007523	<0.000001	1112

**O34833**	<0.000001	0.007364	<0.000001	0.000001	827

**P80877**	<0.000001	>0.99	>0.99	0.000002	892

**P20166**	0.000046	0.272716	0.044373	0.000003	1948

**P94565**	<0.000001	>0.99	0.264667	0.000003	1033

**P19582**	<0.000001	>0.99	0.011528	0.000008	903

**O31709**	<0.000001	>0.99	>0.99	0.000020	295

**O32167**	0.000020	>0.99	0.001374	0.000021	2891

**P25994**	0.000001	0.755894	0.006728	0.000072	1641

**P71044**	0.002703	>0.99	0.000005	0.000284	635

**P21464**	0.000613	>0.99	<0.000001	0.000473	1272

**O32243**	0.000003	0.599332	>0.99	0.000505	404

**O34442**	<0.000001	>0.99	0.047638	0.000552	291

**P35149**	<0.000001	0.000169	>0.99	0.000693	751

**O32157**	0.000035	0.016583	<0.000001	0.000796	957

**P21467**	0.015346	>0.99	0.000001	0.001040	781

**P39912**	0.000327	>0.99	>0.99	0.002252	878

**P23446**	*>0.99*	>0.99	<0.000001	0.002510	451

**P49785**	0.039250	>0.99	0.000245	0.002557	536

**O31567**	0.001315	>0.99	0.000289	0.002616	613

**P46922**	*0.069376*	>0.99	0.000162	0.002782	581

**Q01625**	*0.190501*	>0.99	<0.000001	0.003031	325

**P54537**	*>0.99*	>0.99	<0.000001	0.004345	471

**P71070**	*0.417433*	>0.99	0.000001	0.009605	314

**Q9KWU4**	*0.280189*	>0.99	0.000448	0.009979	807

**O06745**	0.000005	>0.99	0.013199	0.011193	485

**O35007**	0.002474	>0.99	>0.99	0.011411	410

**O05252**	*0.111360*	0.104107	0.601796	0.017767	835

**O32218**	0.009165	>0.99	>0.99	0.018027	567

**O34633**	0.000185	>0.99	>0.99	0.025892	433

**P94421**	*>0.99*	>0.99	<0.000001	0.027798	509

**O32247**	0.025031	>0.99	>0.99	0.028009	845

**P42971**	0.000473	>0.99	>0.99	0.032372	475

**P23447**	*0.078522*	>0.99	0.721913	0.036485	459

**P09339**	0.000203	0.244699	>0.99	0.040871	619

**P37870**	0.017921	0.000748	>0.99	0.041718	1113

**O32047**	*0.178170*	>0.99	<0.000001	0.042466	665

**P12042**	0.002703	>0.99	>0.99	0.043005	699

**P32399**	0.000267	>0.99	>0.99	0.045315	388

**P08066**	*0.105333*	>0.99	0.000002	0.048719	644

**P40779**	*0.083444*	>0.99	0.053914	0.049061	409

Exemplary shown for experiment A, the table displays all significantly expressed proteins (*p *< 0.05, *p*-value adjustment using Holm) reported by a Kruskal-Wallis rank sum test (further results can be found in the supplementary data). Proteins are sorted by the Kruskal-Wallis' *p*-value in ascending order. In summary, 64 proteins were found with a significant change in their abundance. By comparison, 52 of them were also reported as significant by the ANOVA (not significant printed in italics).

For experiment B a performed ANOVA identifies 386 proteins as significantly differently regulated with regard to the factor time (see Additional file 2). While a Fligner-Killeen test (ii) states that 30 of these proteins have inhomogeneous variances, in an impressive number of cases (325 proteins) a violation of the normal distribution assumption was indicated by the Shapiro-Wilks test (i). In summary, only 61 proteins fulfilled the prerequisites of the ANOVA and can therefore-without hesitation-be declared as significantly differentially regulated. Applying in contrast the non-parametric Kruskal-Wallis rank sum test, even 493 proteins reveal significant changes in their abundance between the five time points. Neglecting the requirements of the ANOVA, the agreement between both approaches is higher than 90% and counts 355 differentially regulated proteins.

In the third experiment C, a comparably small number of only 17 proteins was declared significant by the ANOVA (see Additional file 3). Here, no protein showed any inhomogeneous variances (ii), and only in one case the normal distribution assumption was violated (i). The null hypothesis of no differential regulation was rejected for 10 proteins by the Kruskal-Wallis test, which without any exception were also in the result set of the ANOVA.

To determine a general measure of conformity, resulting *p*-values of the ANOVA and the Kruskal-Wallis test for all proteins were compared using Spearman's rank correlation coefficient [37]. Here, a value of *r *= 0.8290125 for experiment A, *r *= 0.836562 for experiment B, and *r *= 0.7780913 for experiment C was calculated. Following Cohen's rating of *r *≥ 0.5 as a strong correlation [38], in summary, for all experiments a large degree of similarity between both results can be attested.

#### Visualization of statistical tests

**Figure 1:**
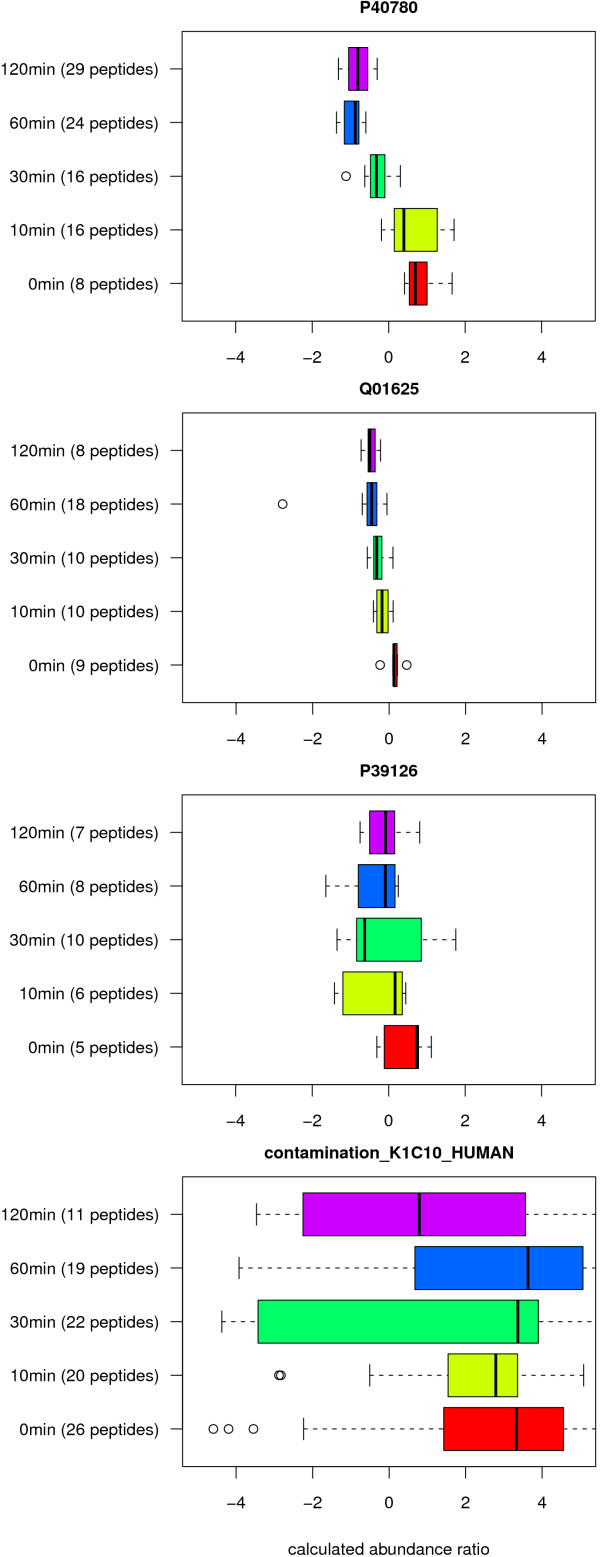
**Box-whisker plot**. Box- and whisker plots provide a simple but also very powerful way to visualize the results of an ANOVA, and give an overview of five essential characteristics of a series of measurements including median, lower and upper quartiles, and extreme values. This figure demonstrates the differences between the calculated abundance ratios of four proteins. Both the ANOVA as well as the Kruskal-Wallis test show a significant change in abundance of the protein P40780 in experiment A. Although the measurements are not following a Gaussian distribution, there is clearly a differential regulation over time. The membrane protein Q01625 reveals only small changes. It was nonetheless regarded significant by the Kruskal-Wallis test, but not by the ANOVA after *p*-value adjustment. P39126, a NADP-dependent dehydrogenase, is not showing any clearly distinguishable and significant pattern of expression. A reason therefore might be a high biological variance but, of course, also technical errors in measurement. Fortunately, the same-albeit to an even greater degree--applies for the human protein K1C10, which is obviously a contamination.

A simple but also very powerful way to visualize the results of an ANOVA and review individual proteins, e. g. if statistical significance is doubtable, are box- and whisker plots [39]. These provide an overview of five essential characteristics of a series of measurements to compare distribution and relative location between different groups. Figure 1 contains four plots that visualize the differences between the calculated abundance ratios of four selected proteins over time. As an example, both the ANOVA as well as the Kruskal-Wallis test show a significant change in abundance of the protein P40780 in experiment A. Although the measurements are not following a Gaussian distribution, there is clearly a differential regulation over time. The membrane protein Q01625 reveals only small changes and was regarded significant by the Kruskal-Wallis test, but not by the ANOVA after *p*-value adjustment. P39126, a NADP-dependent dehydrogenase, is not showing any clearly distinguishable and significant pattern of expression. A reason therefore might be a high biological variance but, of course, also technical errors in measurement. Fortunately, the same-albeit to an even greater degree-applies for the human protein K1C10, which is an obvious contamination.

### Question 2) Identification of co-regulated proteins

Applying cluster analysis on isotope-labeled quantitative proteomics datasets aims to identify proteins that reveal similar patterns of regulation. To this end, the clustering process aggregates those proteins in groups that are characterized by a similar series of measurements. Accordingly, a solution has to be found i) to determine the similarity for two proteins **x **= {*x_i_*, *i *= 1, . . . , *N*} and **y **= {*y_i_*, *i *= 1, . . . , *N*}, and ii) to aggregate clusters from these similarity values, i. e. the formulation of an algorithm. These two problems span the space of algorithmic solutions to the clustering problem. While an answer to question 1 was searched on the peptide level, cluster analysis demands averaging over all calculated peptide abundance ratios to form one value per protein and condition. Being one of the most frequently used statistics for this purpose, here the arithmetic mean was selected, though, also the median or the trimmed mean could have been a good choice. Aiming to achieve utmost accurate analysis results, only those proteins where included having at least two peptide measurements per condition. These are 188 proteins for experiment A, 935 for experiment B, and 196 for experiment C. At this point, we intentionally decided against taking into account more proteins in our analysis as this may have resulted in the necessity to replace missing values in the data. For experiment A, this was exemplary implemented and tested by replacing any missing value with each protein's mean abundance ratio over all conditions. Allowing for example one missing value per protein in the data, cluster analysis would cover 263 proteins for this experiment. Since further analysis showed comparable clustering results (data shown in QuPE), we refrained, in the following, from including any protein having less than two measurements per conditions.

Given the matrix of protein ratios per condition, a common solution to the clustering problem i) is to apply the Euclidean distance. This can be interpreted as the physical distance between two points, and is, hence, very appealing [20]. Given **x**, **y **this distance *d *is defined as follows:(3)

In some cases, actual differences in the abundance ratios of two proteins are negligible but instead a positive or negative correlation between two proteins is of interest. Under these conditions similarity measures based on correlation such as Pearson's uncentered or centered correlation coefficient may be utilized (see Supplementary material). However, it has to be considered that this method may regard two proteins as similar although one is overly up- and one overly down-regulated.

**Figure 2:**
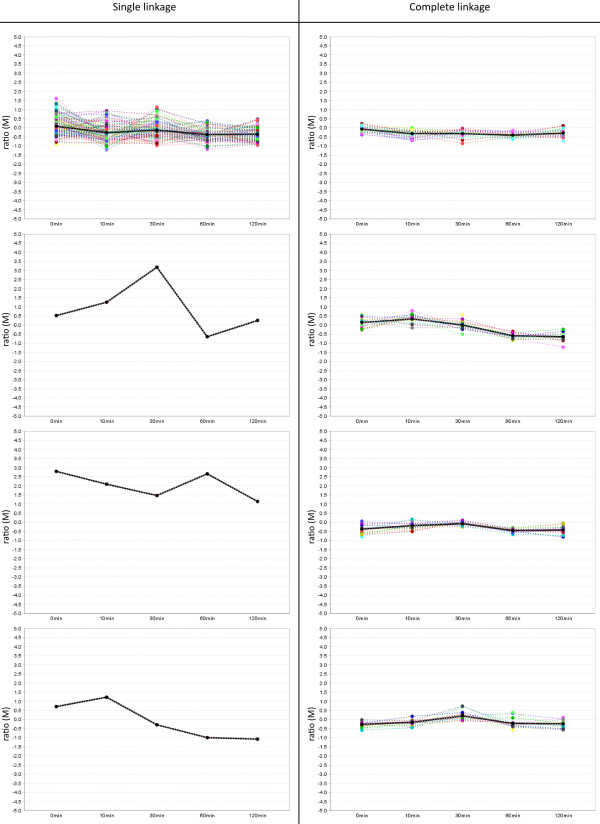
**Comparison of cluster properties - connectedness vs. compactness**. This figure shows each four selected clusters that resulted from two hierarchical cluster analyses using Single-Linkage (on the left side) and Complete-Linkage (on the right side). These results demonstrate the two opposing properties of a clustering connectedness and compactness: Single-Linkage, on the one hand, tends to group as many proteins in a cluster if there is at least a slight similarity. This often leads to one large cluster that contains most of the proteins while other clusters consist of individual outliers. Complete-Linkage, on the other hand, tries to form compact clusters that contain only those proteins that are utmost similar.

**Figure 3:**
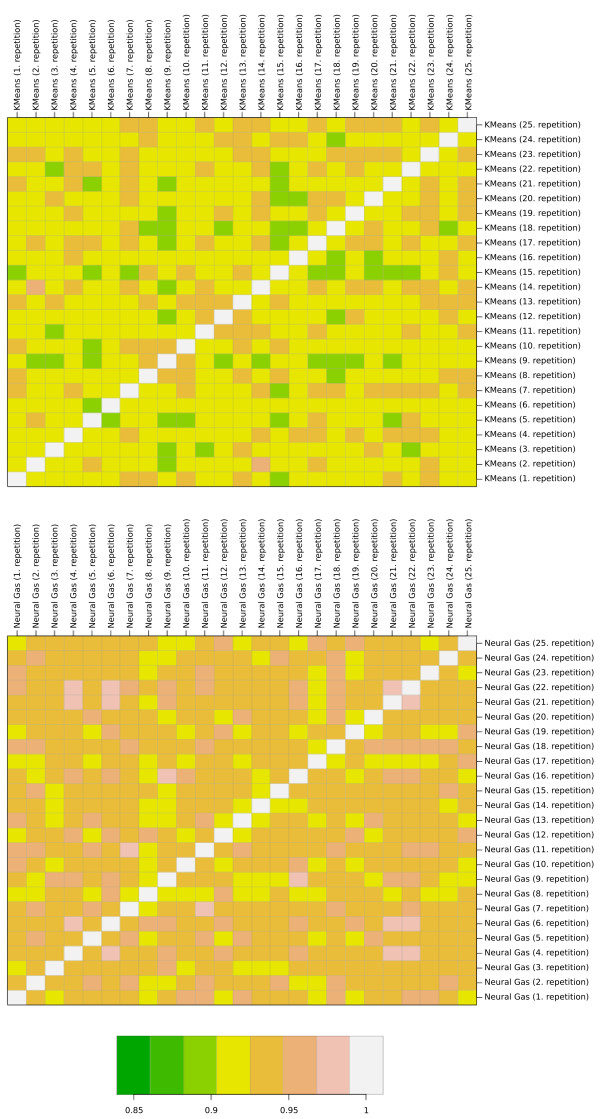
**Comparison of K-means and Neuralgas using the Rand measure**. This figure visualizes the problems of K-means in terms of reproducibility. Both K-means and Neuralgas were executed 25 times with a fixed cluster number of 20. In each repetition, the initial cluster centers were randomly sampled from the input dataset. A pairwise degree of similarity between each two clustering results was then computed using the Rand index. A value of 0.0 indicates no similarity, while a value of 1.0 means identity. The shown heatmaps visualize this measure. The upper part of this figure displays the results of K-means, the lower part those of Neuralgas. In all cases the outcomes of two invocations of both algorithms are slightly dissimilar, and not identical. However, the K-means approach reveals a significant higher variance in its results.

The cluster algorithm to solve ii) determines how all measurements, i. e. proteins, are to be grouped into clusters. Two opposing properties to characterize a cluster result are connectedness and compactness [27]. Transfered to the context of proteomics this can be seen as the conflict between the two ideas to, on the one hand, combine as many proteins as possible if they reveal only a slight similarity and to form compact clusters that contain only those proteins that are utmost similar, on the other hand (see Figure 2). Hierarchical cluster analysis (HCA) methods (a) organize the input data (i. e. the measurements) into a tree structure exposing the relationships from the most similar to the most different proteins. Using some straightforward criterion (like a horizontal cut through the tree) clusters are generated from the result. Single- and Complete-Linkage are two approaches that represent the aforementioned opposing properties [21]. Average-Linkage can be regarded a compromise of both approaches, and Ward's method is based on the idea that each time two clusters of proteins are combined the variance within this new cluster will increase-an increase that should be as minimal as possible [22,23,40]. In contrast, partitioning cluster algorithms (b) follow an optimization strategy to successively assign each protein of the input dataset to one distinct group. In the outcome, each of these clusters is characterized by a typical representative-its cluster center or profile-allowing for a direct reading of the cluster's mean abundance ratios. The K-means algorithm [24,25], the most prominent member of this group of cluster algorithms, has a clear disadvantage as it strongly depends on the initial definition of these group centers and repeated invocation might therefore yield varying results. Neuralgas claims to be an enhancement as it takes into account a "neighborhood ranking" of all proteins that are assigned to a cluster-an advantage bought by an increase in computational running time [41]. To analyze this problem in terms of reproducibility, both K-means and Neuralgas were executed 25 times with a fixed cluster number of 20. In each repetition, the initial cluster centers were randomly sampled from the input dataset (experiment A). A pairwise degree of similarity between each two clustering results was then computed using the Rand index [42]. Here, a value of *R *= 0.0 indicates no similarity, while *R *= 1.0 means that the results are identical. In all cases the outcomes of two invocations of both algorithms are slightly dissimilar, ranging from *R *= 0.89 to 0.98. In comparison, however, as shown in Figure 3, the K-means approach reveals a significant lower similarity between two results (*p *< 0.001), in other words, a lower reproducibility. Density-based cluster algorithms (c) such as fuzzy C-means [26] allow a fuzzy assignment of each data point/protein to one or more clusters. However, to compare the results to other clusterings, in the end, each protein is assigned to that cluster which it most likely belongs to, i. e. the cluster with maximal membership.

#### Evaluation of cluster algorithms

**Figure 4:**
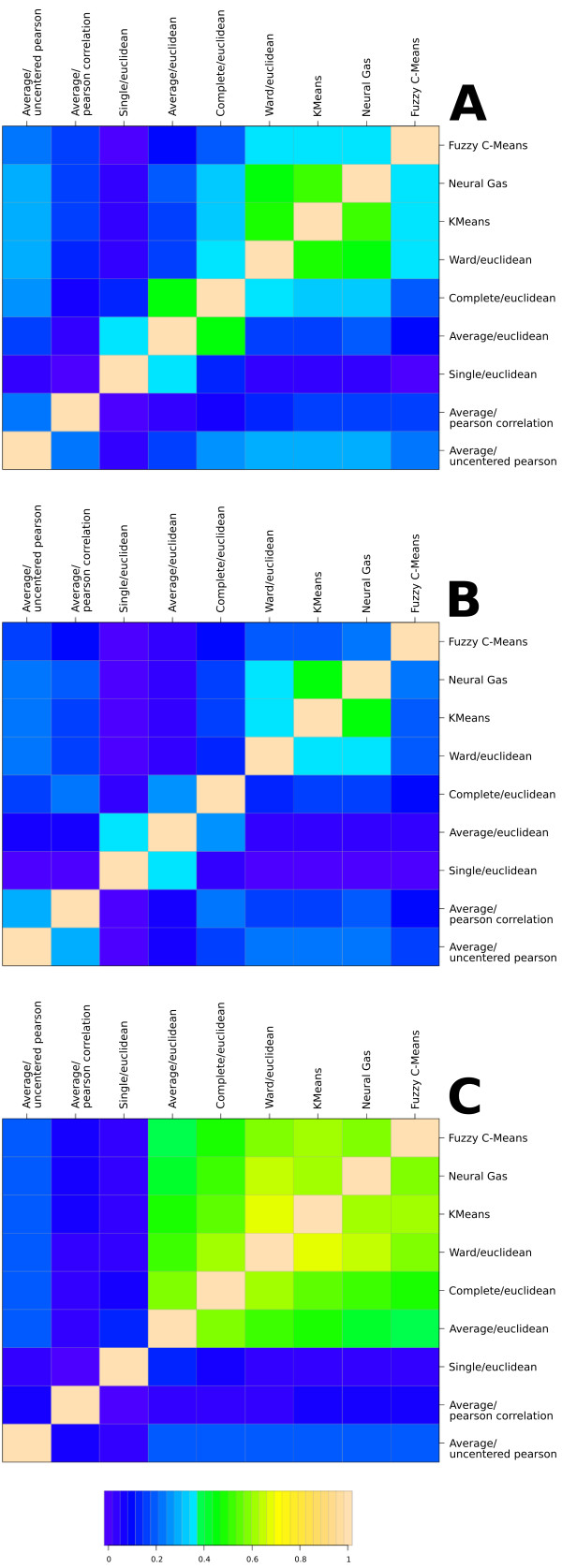
**Comparison of cluster algorithms using the Rand measure**. We estimated a pairwise degree of similarity between two clustering results both with identical cluster numbers produced by two different algorithms using the adjusted Rand index. The three heatmaps shown in this figure each visualize the mean of all Rand indexes computed for cluster numbers from two to 50 for experiment A and C. For experiment B, we selected a different highest cluster number--here 100 was chosen--in respect to the experiment's dataset size and, hence, its higher number of quantified proteins. In all three experiments, notably, a high degree of similarity is observed between the three cluster methods K-means, Neuralgas, and HCA using Ward's Linkage; in experiment C, in addition, to fuzzy C-means, Complete- and less pronounced to Average-Linkage (the two latter with Euclidean distances). In experiments A and B, a slight similarity is, furthermore, found between Single- and Average-Linkage (likewise with Euclidean distances).

Given this plenitude of algorithmic approaches to solve the clustering problem one may ask in how far their outcomes differ, particularly, applied to quantitative proteomics data. Without any interpretation of the resulting clusterings, we therefore estimated a pairwise degree of similarity between two clustering results both with identical cluster numbers produced by two different algorithms. For this purpose, the adjusted Rand measure [42] was utilized. Figure 4 visualizes the mean of all Rand indexes computed for cluster numbers from two to 50 for experiment A and C, and from two to 100 for experiment B. For the latter, we selected a different highest cluster number in respect to the experiment's dataset size and, hence, its increased number of quantified proteins. A strong but not surprising degree of similarity (A/B: *R *> 0.45, C: *R *> 0.6) was found between the two methods K-means and Neuralgas. Furthermore, both methods show a comparably high similarity (up to *R *> 0.6 in experiment C) to HCA using Ward's linkage and Euclidean distances (Ward/Euclidean); in experiment C, in addition, to fuzzy C-means, Complete- and less pronounced to Average-Linkage (the two latter with Euclidean distances). Only in experiments A and C, a pronounced similarity (*R *> 0.45) can be attested to the outcomes of HCA using Complete- and Average-Linkage (Complete/Euclidean, Average/Euclidean). In experiments A and B, a slight similarity is, furthermore, found between Single- and Average-Linkage (likewise with Euclidean distances). On the contrary, it has to be pointed out that methods such as Average-Linkage using correlation-based distances or, with the aforementioned exception, Single-Linkage using Euclidean distances each yielded an entirely unique output. In summary, the results of this comparison (see Supplementary information for further details) demonstrate that the choice for a cluster algorithm is not arbitrary but instead strongly influences the outcome.

**Figure 5:**
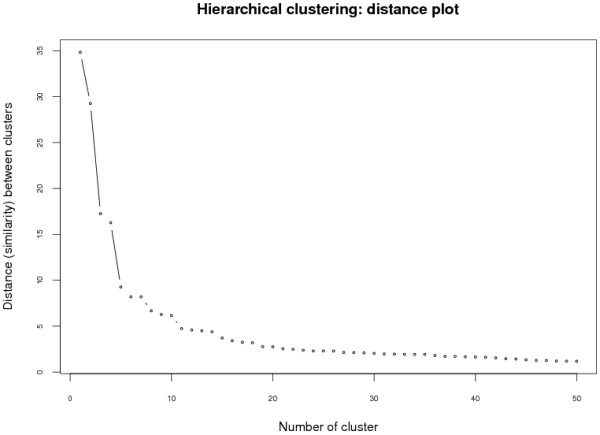
**HCA - distance plot**. In hierarchical cluster analysis, a simple but powerful way to assess the "true" number of clusters of a dataset is a visual analysis of each possible cluster number set in relation to the distance (similarity) between two clusters that will be merged. An optimal solution can be identified by searching a "knee" in the plot. In this example this can be identified around the 10-cluster solution.

From a computational point of view, a number of quality measures have been proposed to evaluate and rank the outcomes of cluster algorithms. Because of opposing characteristics such as compactness and connectedness, however, no definite criteria can be formulated that describes an optimal clustering of a dataset. This pertains not only to the applied cluster algorithm but also to the "true" number of clusters of a dataset. Proposed measures that base solely on the clustering itself and the underlying dataset [27] range from early approaches [43-45] up to novel instruments [46,47] (see Additional file 4 for further details). In hierarchical cluster analysis, a simple but powerful way to assess the "true" number of clusters of a dataset is a visual analysis of each possible cluster number set in relation to the distance (similarity) between the two clusters that are merged to gain a clustering of this size. An optimal solution can be identified by searching a knee in the plot (see Figure 5 for an example).

From a biological point of view, a good cluster solution is much more difficult to assess. In general, this demands additional knowledge about the proteins under investigation, e. g. a set of known class labels or a previously determined analysis result. A calculated clustering could then be compared to the labels to determine a degree of similarity. In real life experiments this information is, however, rarely available for all analyzed proteins. An automatic evaluation based on external information is, hence, nearly impossible. Nevertheless, biologically meaningful clusters are characterized by consisting of proteins that belong to a similar functional category or which are involved in the same metabolic pathway.

**Figure 6:**
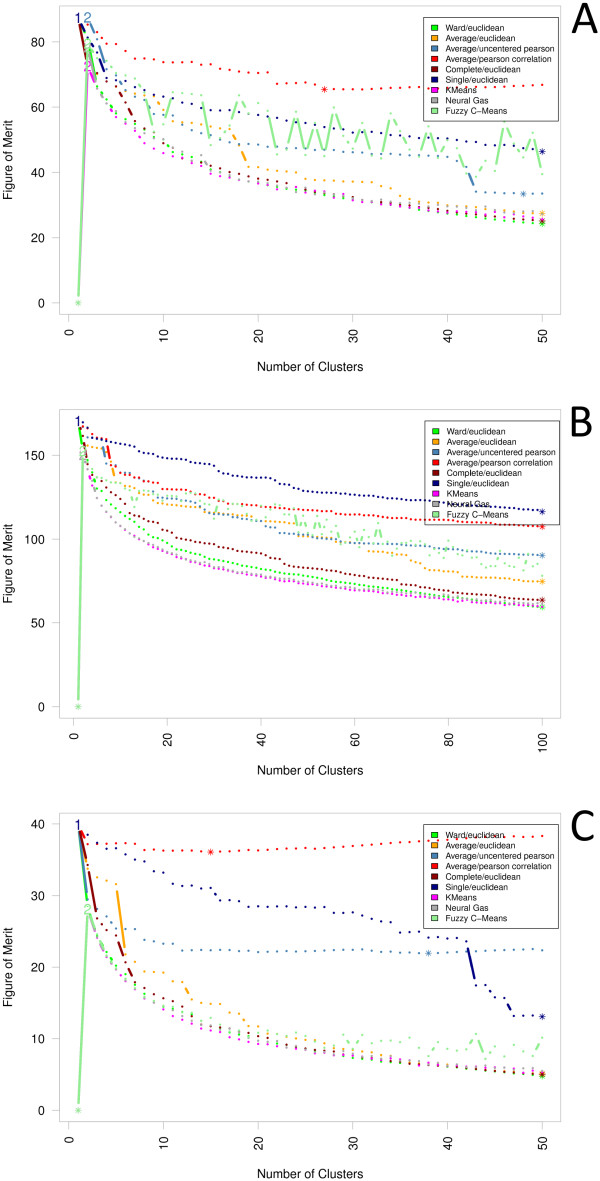
**Figure of Merit**. Figure of Merit (FOM) has been delineated by Yeung *et al*. [46] particularly for the analysis of gene expression data. The index estimates the predictive power of a cluster algorithm, and thereby revealed HCA using Ward's linkage and Euclidean distances, K-means as well as Neuralgas as the best performing cluster algorithms, while correlation-based cluster algorithms, Single-Linkage using Euclidean distances, and--at least in two experiments--fuzzy C-means produce the least reliable results. The lowest observed value is marked with an asterisk symbol, whereas at the position of the highest index value the corresponding cluster number is printed.

Assistance in choosing a cluster algorithm, particularly, for the analysis of gene expression data, was recently offered by Yeung *et al*. [46]. They delineated an instrument called Figure of Merit (FOM) to evaluate cluster solutions. The idea of their method is to integrate a kind of bootstrapping approach (cf. [18]), and thereby to estimate the predictive power of a cluster algorithm. Applied on our data, this index revealed Ward/Euclidean, K-means as well as Neuralgas as the best performing cluster algorithms, while correlation-based cluster algorithms, Single-Linkage using Euclidean distances, and-at least in two experiments-fuzzy C-means produce the least reliable results (see Figure 6).

**Figure 7:**
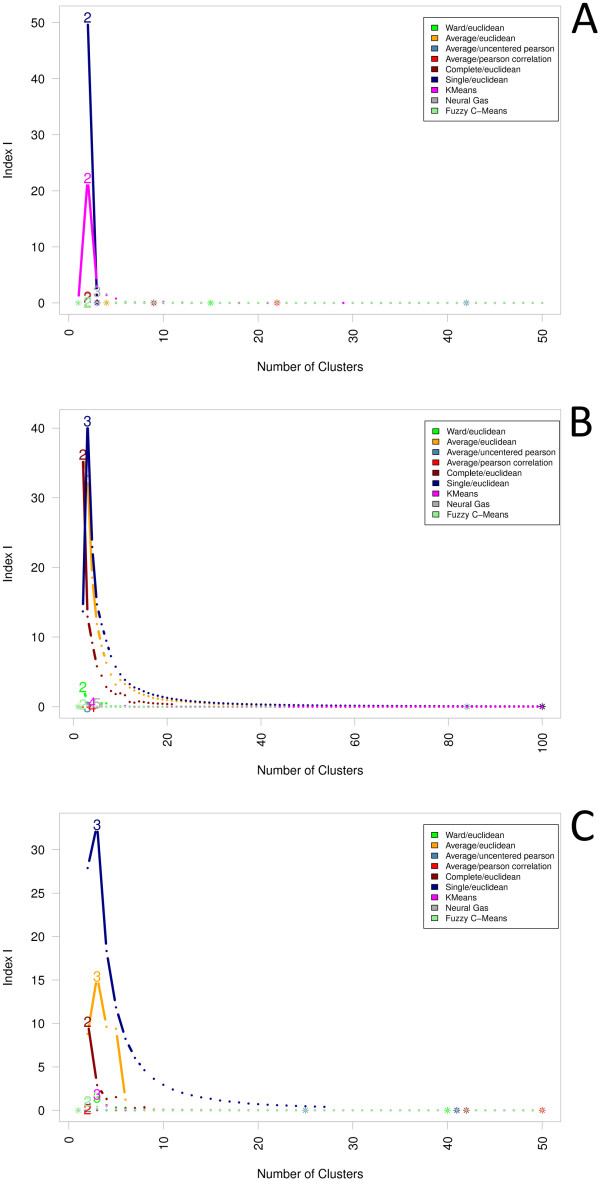
**Index I**. The cluster index "Index I" tends to favor smaller cluster numbers between two and three clusters. From a computational point of view this is clearly a good result. Unfortunately, from a biological point this does not allow any meaningful interpretation of the data. In general, these small clusterings only characterize individual outliers, while the rest of the clusters are found with a high number of cluster members having everything clustered together that reveals only a slight similarity.

**Figure 8:**
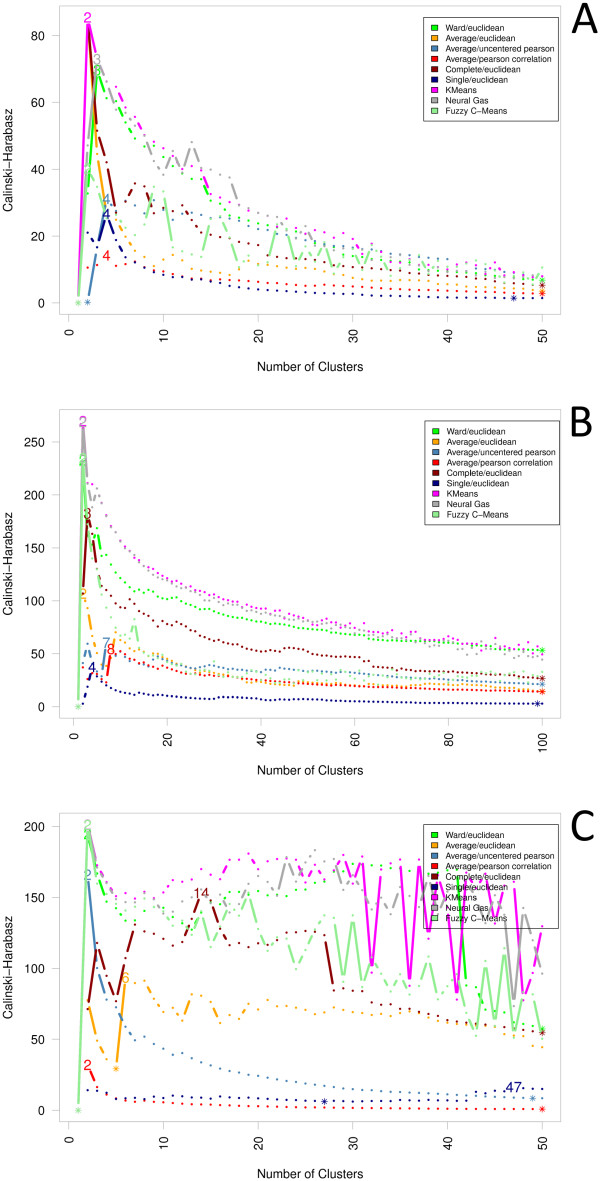
**Calinski-Harabasz**. Similar to the "Index I" the cluster index of Calinski and Harabasz tends to favor smaller cluster numbers between three and four clusters. In the same manner, the applicability with respect to the biological question also remains questionable.

Aiming to determine an optimal clustering of each proteomics dataset regarding both the biological as well as the computational point of view, we analyzed the results of all applied cluster algorithms using a diversified selection of cluster indexes. Here, the index of Calinski and Harabasz [43], which sets the similarity of all proteins grouping together in a cluster in relation to the dissimilarities of each two clusters, and even more the Index I [47], which follows a comparable approach, tend to favor smaller cluster numbers between two and three clusters (see Figures 7 and Figure 8; Additional files 5, 6 and 7 for further details). While from a computational point of view these results seem reasonable, from a biological point of view they do not allow any meaningful interpretation of the data. In general, these small clusterings only characterize individual outliers, while the rest of the clusters are found with a high number of cluster members having everything clustered together that reveals only a slight similarity. Experiment C is, in some respect, an exception as here the cluster index of Calinski and Harabasz gives evidence for higher cluster numbers, e. g. 14 for Complete/Euclidean. This could result from the fact that the data of this experiment has a comparably low dimensionality as there are only two different abundance ratios per protein-one for growth on benzoate, one for glucose.

**Figure 9:**
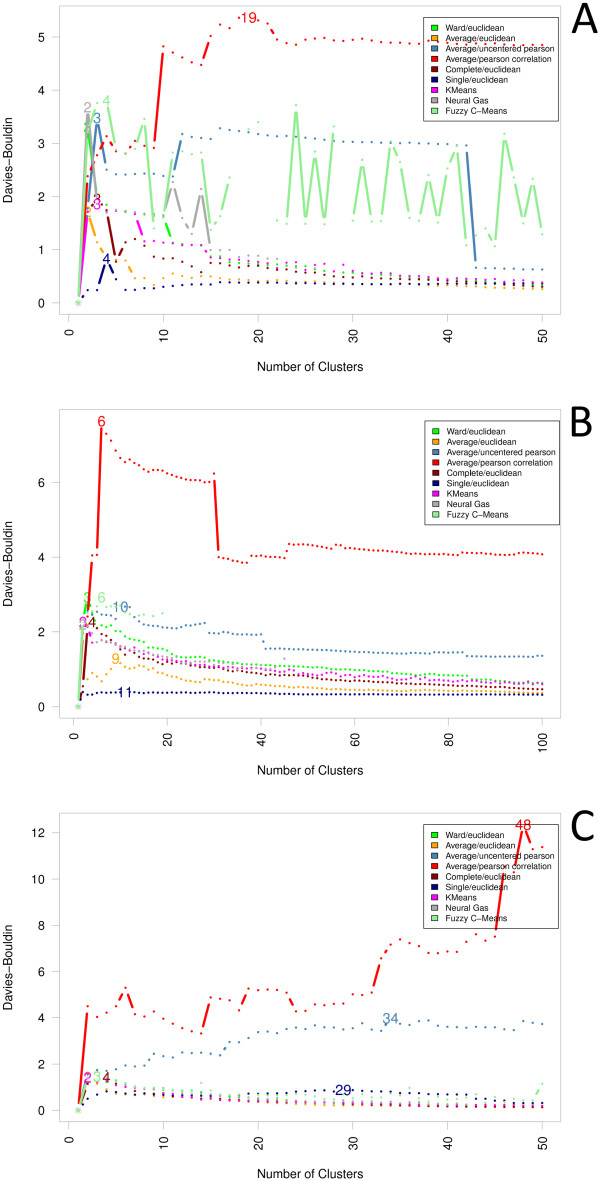
**Davies and Bouldin**. Instead of simply proposing a cluster index, Davies and Bouldin formulated a general framework for the evaluation of the outcomes of cluster algorithms. In contrast to other indexes, an optimal cluster solution is indicated by the minimal calculated index value. For instance, for the two cluster algorithms K-means and Neuralgas a local minimum can be located around the 30-cluster solution. A general interpretation of this index, however, seems to be difficult due to a strong tendency towards constantly decreasing index values with regard to large cluster numbers.

Davies and Bouldin formulated a general framework for the evaluation of the outcomes of cluster algorithms [44]. An instance of their index provided by Halkidi *et*. *al *[28] follows the idea that an optimal solution to the clustering problem has been found as soon as for each cluster no other utmost similar cluster-with regard to the intra-cluster error sum of squares as well as the distance between clusters-can be identified. In contrast to other indexes, this is indicated by the minimal calculated index value (see Figure 9). In experiment A, for instance, for the two cluster algorithms K-means and Neuralgas, a local minimum can be located around the 30-cluster solution. A general interpretation of this index, however, seems to be difficult due to a strong tendency towards constantly decreasing index values with regard to large cluster numbers. An exception are both correlation-based cluster algorithms (Average/Pearson correlation, Average/Uncentered Pearson): at least for experiment C, index values seem constantly to increase providing nevertheless no clear statement with regard to an optimal clustering of the data.

**Figure 10:**
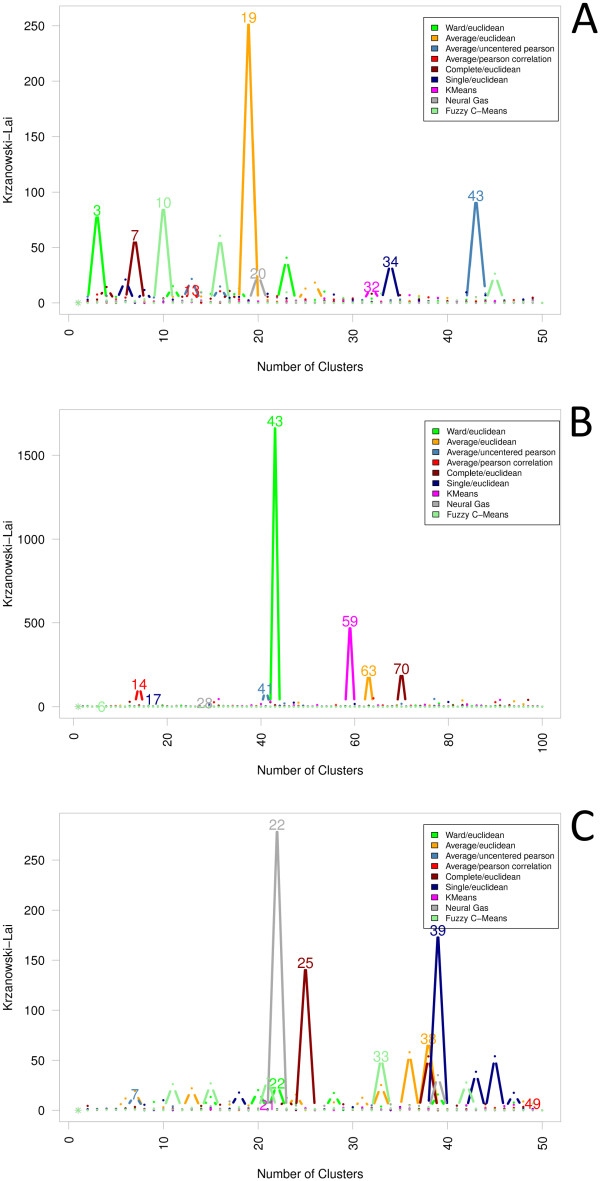
**Krzanowski and Lai**. The cluster index of Krzanowski and Lai showed both from a biological as well as from a computational point of view meaningful cluster numbers: for the data of experiment A, there were found cluster numbers between 3 for Ward/Euclidean--here a second local maximum was found at 23 clusters--and 43 for Average/Uncentered Pearson as the true clustering of the data; for experiment B, between 14 (Average/Pearson correlation) and 70 (Complete/Euclidean), with a protruding 43-cluster solution applying Ward/Euclidean; and for experiment C, e. g. at seven clusters for Average/Uncentered Pearson, 22 for Ward/Euclidean and 38 for Average/Euclidean.

We draw conclusions differing from that obtained in a microarray study [48], when we investigated the index of Krzanowski and Lai [45]. In that study-a comparison of five cluster measures on six different microarray datasets-the index revealed a poor performance in terms of predictive power. However, in our analysis the application showed both from a biological as well as from a computational point of view meaningful results (see Figure 10). For our proteomics dataset of experiment A, the index suggested a cluster number between three (Ward/Euclidean), which also shows a local maximum at 23 clusters, and 43 clusters (Average/Uncentered Pearson). To extend our knowledge about the identified proteins, information from COG (clusters of orthologous groups of proteins) [49] and the Kyoto Encyclopedia of Genes and Genomes (KEGG) [50] was integrated. Looking at the 23-cluster solution produced by Ward/Euclidean in detail, the outcome reveals a reasonable biological finding. It consists of several clusters of proteins sharing a common function, e. g. regarding cell wall biogenesis, metabolism of amino acids, or motility and chemotaxis, and corresponds to the findings of Hahne *et al*. [1]. Proteins that reveal a similar pattern of regulation are for example eight proteins that are involved in amino acid transport and metabolism. The proteins in this cluster appeared down-regulated after 30 minutes. In another cluster eight proteins, which are mostly responsible for cell motility, show an increase in their relative abundance over time.

For experiment B, the index of Krzanowski and Lai displays cluster numbers between 14 (Average/Pearson correlation) and 70 (Complete/Euclidean), whereby, inter alia, a 43-cluster solution for HCA using Ward and Euclidean distances sparked our interests. This solution distinguishes several groups of proteins according to their different regulation during the time course. Analogous to the results of Otto *et al*. [2] a number of proteins were found with decreasing abundance ratios after cells entered stationary phase. These are, presumably, subjected to degradation. The resulting clustering included a group of 31 proteins, which play a role in the metabolism of nucleotides and amino acids; a cluster of 10 proteins similarly involved in secondary metabolites biosynthesis, transport and catabolism; and another cluster with 20 functionally related proteins with regard to amino acid transport and metabolism. On the opposite, a cluster could be identified with 10 proteins strongly increasing in amount after the transition from exponential to stationary growth phase. Specifically, these are, for example, the proteins O34425 and P54418, which were also highlighted as significantly differentially regulated in the original publication.

Meaningful results where also observed in the application of the index of Krzanowski and Lai on the data of experiment C. Here, an optimal clustering was found for example at seven clusters for Average/Uncentered Pearson, 22 clusters for Ward/Euclidean and 38 clusters for Average/Euclidean. In the 22-cluster solution using Ward/Euclidean a number of ribosomal proteins showed no change in regulation due to the two different growth media. In contrast, proteins belonging to the COG functional categories amino acid transport and metabolism, and energy production were down-regulated during growth on benzoate including for example Cg1806, an enzyme involved in sulfur metabolism.

#### Visualization of cluster results

**Figure 11:**
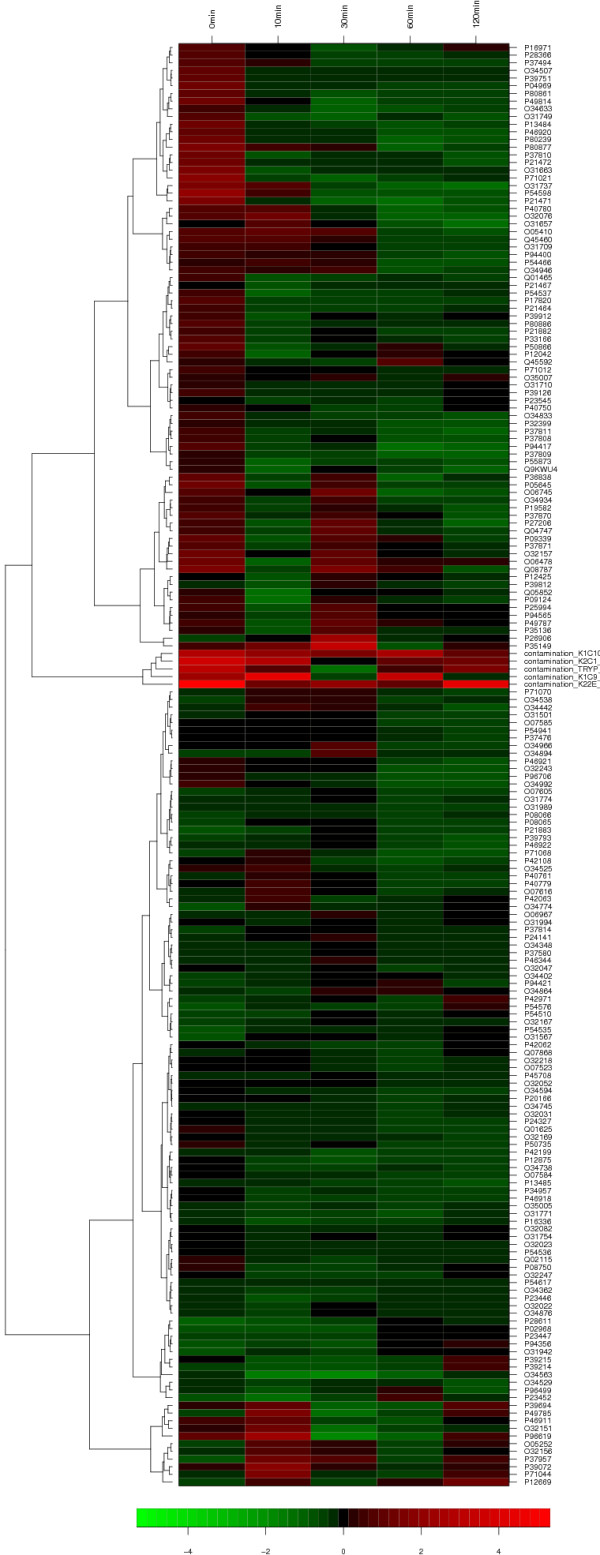
**Heatmap**. This figure shows the results of a hierarchical cluster analysis on the data of experiment A using Ward's linkage method and Euclidean distances in form of a heatmap. The columns of the heatmap indicate the five different timepoints, that the samples have been taken at, while each row stands for a protein.

A typical visualization of the results of a hierarchical cluster analysis is a heatmap as exemplary shown in Figure 11[51]. Calculated abundance ratios are color coded. An attached dendrogram reveals the hierarchical relations between the proteins. In many cases, one is not interested in determining the relationships between all proteins, but instead of representative groups of proteins that show a very similar pattern of regulation. Here, a simple XY-plot may provide an adequate visualization.

## Conclusions

This work aims at paving a straight path through the jungle of computational methods to analyze mass spectrometry-based isotope-labeled datasets, targeting the two questions that typically arise in proteomics experiments: 1) which proteins are differentially regulated regarding the selected experimental conditions, and 2) are there groups of proteins that show similar abundance ratios, indicating that they have a similar turnover? In contrast to other types of Omics experiments, mass spectrometry-based proteomics is faced with particular challenges: due to background signals in mass spectra the data is for example comparatively noisy, and, because of unidentified peptides, values are missing from the measurements [52]. To take these problems into account, we developed our evaluation strategy based on three recently published datasets on *Bacillus subtilis *and *Corynebacterium glutamicum*. In an ideal situation, we would expect that two commonly applied tests to answer question one reveal the same proteins as significantly differentially regulated, and indeed, there was found a strong congruence between the outcomes of an ANOVA and a Kruskal-Wallis rank sum test. However, an ANOVA, strictly speaking, in many cases could not be evaluated, because the normal distribution assumption was often not fulfilled. "Asking whether ANOVA [...] assumptions are satisfied is not idle curiosity. The assumptions of most mathematical models are always false to a greater or lesser extent. The relevant question is not whether ANOVA assumptions are met exactly, but rather whether the plausible violations of the assumptions have serious consequences on the validity of probability statements based on the standard assumptions." [[53], p.237]. As an example, differences in the abundance ratios of the protein P40780 (experiment A, see Figure 1) suggest that, in this case, the normal distribution assumption may be negligible. In conclusion, we recommend to firstly rely on the results of an ANOVA, but secondly, to always take into consideration Kruskal-Wallis. Results should then be compared and further visually investigated using for example Box- and Whisker-plots. In all tests, because of the multiple testing situation, adjustment of computed *p*-value should take place.

Question two is even harder to answer: With the aim of producing biologically meaningful results, we are clearly interested in grouping those proteins in a cluster that reveal an utmost similar pattern of abundance ratios in our experiment. Hence, Single-linkage is not applicable for this purpose, which is also proven by the development of the Figure of Merit. If the benefits of a hierarchical cluster analysis are requested, Ward's method has proven a good choice. If there isn't, Neuralgas should be selected, which clearly outperforms the K-means approach, in particular, regarding the reproducibility of its results. The only drawback of this algorithm might be its comparatively high computational complexity, which is, however, negligible taken into consideration today's average computing resources. In our application study, we found-from a biological point of view-interesting clusters of proteins that both revealed a similar pattern of regulation and fulfilled a similar biological function using these two approaches.

Correlation-based distance measures should only be applied if they can be justified by the underlying experimental hypotheses, e. g. if proteins are expected to be commonly regulated but not at an equal level of abundance. The most difficult part is the validation of a cluster result to gain the "true" number of clusters of a dataset. Here, the cluster index of Krzanowski and Lai turned out to produce both computationally as well as biologically meaningful results. In contrast to other investigated validity measures the index solely relies on the internal compactness of clusters, which seems to correspond to our objective of clustering those proteins that reveal a highly similar pattern of regulation.

To further evaluate cluster analysis results, we recommend including annotation data, such as functional categories. If for example a cluster analysis reveals a group of proteins similarly regulated that furthermore also fulfill a similar role in the cell metabolism, the clustering result can certainly be regarded as more meaningful.

All analyses were performed using the rich internet application QuPE. Results as well as datasets are available online at http://qupe.cebitec.uni-bielefeld.de (see Additional file 8 for a short guide through the data).

## Methods

### Proteomics datasets

For the evaluation and comparison of the different statistical analysis methods we have chosen three different datasets. The first experiment (A) was conducted by Hahne *et al*. [1]. In a study on *Bacillus subtilis *wildtype strain 168 (*trpC2*) the adaption of the organism to salt stress was analyzed at the level of the proteome as well as the transcriptome. Each three samples were grown in ^15^N-labeled medium and mixed with equal amounts of unlabeled, so to say ^14^N-labeled, proteins for relative quantification. LC-MS/MS measurements on an LTQ Orbitrap XL (Thermo Fisher Scientific, Bremen) coupled to a nanoAcquity UPLC (Waters) resulted in each 60 raw data files, which were then transformed into the open source format mzXML using the tool "ReAdW" [54,55]. It has to be noted that in our experiment only the membrane fraction was investigated, which however also comprises high numbers of cytosolic proteins (>70%, [1]).

Likewise targeting *Bacillus subtilis*, Otto *et al*. performed a comprehensive monitoring of temporal changes in the proteome, the transcriptome and the metabolome as a result of glucose starvation. In this second experiment (B), sample preparation and labeling have been carried out analogous to A, and the experiment also consists of three replicates. Here, only the cytosolic fraction was included in our analysis, which, nonetheless, has the impressive amount of overall 292 raw data files [2].

The third experiment (C) scrutinizes the physiological adaption of *Corynebacterium glutamicum *to benzoate and glucose each as sole carbon source. Haußmann *et al*. [3] originally performed SIMPLE [56] digest and MudPIT in combination with metabolic labeling using ^15^N on three replicates and comprehensively investigated the membrane proteome. In this work, however, only one replicate of the predigest fraction was taken into account to demonstrate the applicability of the provided evaluation strategy on smaller datasets. Overall, 22 LC-MS/MS runs were considered, all measured using a Accela gradient HPLC pump system coupled to an LTQ Orbitrap (Thermo Fisher Scientific, Bremen).

#### Identification

In contrast to the published work, for experiment A and B data was imported into the rich internet application QuPE [4]. A Mascot (TM) [29] search was conducted using a database that contained the complete proteome of *Bacillus subtilis *as well as an equally-sized set of randomized amino acid sequences allowing for the later calculation of false discovery rates as suggested by Reidegeld *et al*. [57]. Peptide tolerance was set to 10.0 ppm, ms/ms tolerance to 1000.0 mmu, and two missed cleavage sites were allowed. Oxidation of methionine was allowed as a variable modification, and furthermore, a modification of arginine and lysine was introduced to account for a possible selected non-monoisotopic peak of a ^15^N-labeled precursor with a weight of approximately 1 Da [58]. Only hits having a score above Mascot's own significance threshold (*p *< 0.05) were kept. In addition, false discovery rates were calculated in QuPE and required to be below *p *< 0.05. For each protein at least two peptide hits had to be available, and for each spectrum only one, the best-scoring, hit was selected. In experiment A this resulted in 173,044 peptide hits accounting for overall 1445 proteins. The high number of 620,305 identified peptides was found for experiment B. These constitute 2472 different proteins.

For experiment C, protein identification was based on the original Sequest (TM) [30] search results. The database contained 3058 sequences of *Corynebacterium glutamicum*. Filter criteria (for further details please refer to the original publication) were selected in such a way that a false discovery rate of less than 1% was achieved. In summary, 12,870 peptide identifications were imported in QuPE which in turn represent 712 proteins.

#### Quantification

For all three experiments, quantification was performed using QuPE's built-in algorithm using an ^15^N incorporation level of 98% and under consideration of a peptide's elution in a range of 30 to 60 seconds before and after the scan it was identified in. Rather strict parameters were employed (*r *> 0.4, isotopic distribution similarity >0.8) and results were filtered for a signal-to-noise value of at least 3.0. In summary, for experiment A 58,895 peptides could be quantified accounting for 1285 proteins; in experiment B it were 180,913 peptides amounting to 2321 proteins, and in experiment C 3,699 peptides and 589 proteins. In this regard, one special case has to be highlighted as protein identification in the samples of experiment A and B also took into account contaminations by using not only a *Bacillus subtilis *sequence database but also a set of common laboratory contaminants. Obviously, these proteins were not subject to the labeling, but some showed high signal-to-noise values for the unlabeled peptide. We kept these-actually senseless-proteins in our analysis as they provide a good example for measurements having a high variance. Due to a label swap (control ^15^N, experiment ^14^N) in one of the samples not only very high but also very low ratios were obtained.

## Competing interests

The authors declare that they have no competing interests.

## Authors' contributions

SPA performed the evaluation and implemented all methods within the rich internet application QPE. HH, AO, UH, AP and DB provided datasets and material, and contributed to the biological background. TWN and AG initiated, supervised, and directed the project. All authors have read and approved the manuscript.

## Supplementary Material

Additional file 1 **Statistical analysis**. Table showing the ANOVA and Kruskal-Wallis test results of all proteins. Proteins are sorted by the ANOVA's *p*-value in ascending order. The assumption that all values are derived from a normal distribution was investigated using a Shapiro-Wilks test. To analyze the homogeneity of variances of each group a Fligner-Killeen test was performed. All computed *p*-values where corrected using the method described by Holm (experiment A). Click here for file

Additional file 2 **Statistical analysis**. Table showing the ANOVA and Kruskal-Wallis test results of all proteins. Proteins are sorted by the ANOVA's *p*-value in ascending order. The assumption that all values are derived from a normal distribution was investigated using a Shapiro-Wilks test. To analyze the homogeneity of variances of each group a Fligner-Killeen test was performed. All computed *p*-values where corrected using the method described by Holm (experiment B). Click here for file

Additional file 3 **Statistical analysis**. Table showing the ANOVA and Kruskal-Wallis test results of all proteins. Proteins are sorted by the ANOVA's *p*-value in ascending order. The assumption that all values are derived from a normal distribution was investigated using a Shapiro-Wilks test. To analyze the homogeneity of variances of each group a Fligner-Killeen test was performed. All computed *p*-values where corrected using the method described by Holm (experiment C). Click here for file

Additional file 4 **Cluster analysis and cluster validation**. Formal definition of cluster analysis, correlation-based distances and utilized cluster indexes for cluster validation. Click here for file

Additional file 5 **Cluster validation**. Comparison of clustering results having equally sized partitions produced by different cluster algorithms using the adjusted Rand index (experiment A). Click here for file

Additional file 6 **Cluster validation**. Comparison of clustering results having equally sized partitions produced by different cluster algorithms using the adjusted Rand index (experiment B). Click here for file

Additional file 7 **Cluster validation**. Comparison of clustering results having equally sized partitions produced by different cluster algorithms using the adjusted Rand index (experiment C). Click here for file

Additional file 8 **A walk through QuPE**. A walk-through guide introducing some of the functionality of the rich internet application QuPE. Click here for file
