# Supplementary material for: A guide through the computational analysis of isotope-labeled mass spectrometry-based quantitative proteomics data: an application study
Source: Proteome Sci. 2011 Jun 11;9:30. doi: 10.1186/1477-5956-9-30 (PMC3142201; doi:10.1186/1477-5956-9-30)
Supplement: Additional file 4 — Cluster analysis and cluster validation. Formal definition of cluster analysis, correlation-based distances and utilized cluster indexes for cluster validation. [file 1477-5956-9-30-S4.pdf]

Cluster analysis and cluster validation

Correlation-based distances

Apart from metric distances correlation coefficients may be utilized as measures of similarity. Given two measurements $\mathbf{x} = \{x_i, i = 1, \ldots, N\}$ and $\mathbf{y} = \{y_i, i = 1, \ldots, N\}$, Pearson's correlation coefficient is calculated using the following formula (cf. [1]):

$$cor(\mathbf{x}, \mathbf{y}) = \frac{\sum_{i=1}^{n} (x_i - \bar{\mathbf{x}})(y_i - \bar{\mathbf{y}})}{\sqrt{\sum_{i=1}^{n} (x_i - \bar{\mathbf{x}})^2} \sqrt{\sum_{i=1}^{n} (y_i - \bar{\mathbf{y}})^2}} \tag{1}$$

where

$$\bar{\mathbf{x}} = \frac{1}{n} \sum_{i=1}^{n} x_i \tag{2}$$

denotes the arithmetic mean of vector $\mathbf{x}$. Assuming a linear interrelation between the series of measurements of two proteins the correlation coefficient measures their degree of correlation. Resulting values ($[-1 \ldots 1]$) can then be transformed in a distance value d:

$$d(\mathbf{x}, \mathbf{y}) = 1 - cor(\mathbf{x}, \mathbf{y})^2 \tag{3}$$

With a slight modification—the subtraction of each protein's mean abundance value is omitted—Pearson's uncentered correlation coefficient provides another possibility to measure similarities between two classification objects:

$$r(\mathbf{x}, \mathbf{y}) = \frac{\sum_{i=1}^{n} (x_i)(y_i)}{\sqrt{\sum_{i=1}^{n} (x_i)^2} \sqrt{\sum_{i=1}^{n} (y_i)^2}} \tag{4}$$

Formal definition of cluster analysis

Formally, a cluster analysis can be described as the partitioning of a number N of classification objects or—in the sense of proteomics—a number of patterns with an endless dimension P in K groups or clusters $\{C_k, k = 1, \ldots, K\}$. Given N objects $\mathbf{X} = \{\mathbf{x}_i, i = 1, \ldots, N\}$, where $x_{i,j}$ denotes the j-th element of $\mathbf{x}_i$, the grouping of all objects with index $i = 1, \ldots, N$ in clusters $k = 1, \ldots, K$ can be defined as follows:

$$w_{k,i} = \begin{cases} 1, \text{if pattern } \mathbf{x}_i \in \text{cluster } C_k \\ 0, \text{ otherwise} \end{cases} \tag{5}$$

Two conditions apply for the matrix $\mathbf{W}(\mathbf{X}) = [w_{k,i}]_{K \times N}$ to ensure that the association of each object to a cluster is unique (please note that this only applies for hierarchical (a) and partitioning (b) cluster analysis. In case of probabilistic (c) approaches a pattern may belong to more than one cluster with a certain probability):

$$w_{k,i} \in \{0, 1\} \; ; \; \sum_{k=1}^{K} w_{k,i} = 1 \tag{6}$$

Furthermore, let the following definition denominate the number of objects belonging to a cluster C_k:

$$|C_k| = \sum_{i=1}^{N} w_{k,i} \tag{7}$$

Cluster indexes for cluster validation

Calinski-Harabasz

The cluster index of Calinski and Harabasz [2] is calculated using the following equation:

$$CH(K) = \frac{[\text{trace } \mathbf{B} \big/ K-1]}{[\text{trace } \mathbf{W} \big/ N-K]} \quad \text{for} \quad K \in \mathbb{N} \tag{8}$$

where $\mathbf{B}$ denotes the error sum of squares between different clusters (inter-cluster)

$$\text{trace } \mathbf{B} = \sum_{k=1}^{K} |C_k| \parallel \overline{C}_k - \overline{x} \parallel^2 \tag{9}$$

and $\mathbf{W}$ the squared differences of all objects in a cluster from their respective cluster center (intra-cluster)

$$\text{trace } \mathbf{W} = \sum_{k=1}^{K} \sum_{i=1}^{N} w_{k,i} \parallel x_i - \overline{C_k} \parallel^2 \tag{10}$$

Calculated for each possible cluster solution the maximal achieved index value indicates the best clustering of the data. The important characteristic of the index is the fact that on the one hand *trace* $\mathbf{W}$ will start at a comparably large value. With increasing number of clusters K, approaching the optimal clustering solution in K^* groups, the value should significantly decrease due to an increasing compactness of each cluster. As soon as the optimal solution is exceeded an increase in compactness and thereby a decrease in value might still occur; this decrease, however, should be notably smaller. On the other hand, *trace* $\mathbf{T}$ should behave in the opposite direction, getting higher as the number of clusters K increases, but should also reveal a kind of softening in its rise if K gets larger than K^*.

Index-I

Maulik and Bandyopadhyay [3] proposed a cluster index that is, in principle, composed of three individual elements:

$$I(K) = \left(\frac{1}{K} \times \frac{E_1}{E_K} \times D_K \right)^p \quad \text{for} \quad p, K \in \mathbb{N} \tag{11}$$

While the first factor simply normalizes each index value by the overall number of clusters K, the second term sets the overall error sum of squares of the complete datasets in relation to the intra-cluster error of a given clustering:

$$E_K = \sum_{k=1}^{K} \sum_{i=1}^{N} w_{k,i} \parallel x_i - \bar{x}_k \parallel \quad \text{for} \quad K \in \mathbb{N} \tag{12}$$

A third factor takes into account the maximally observed difference between two of the K clusters:

$$D_K = \max_{p,q=1,\dots,K \wedge p \neq q} \parallel \bar{x}_p - \bar{x}_q \parallel \quad \text{for} \quad K \in \mathbb{N} \tag{13}$$

The index computation includes a variable parameter $p \in \mathbb{N}$ that may be “used to control the contrast between the different cluster configurations”’ [3, p.1651]. The authors recommend a value of $p = 2$.

Davies-Bouldin

Instead of simply proposing a cluster index, Davies and Bouldin [4] formulated a general framework for the evaluation of the outcomes of cluster algorithms. In analogy to Halkidi et al. [5] an instance of their index $DB(K)$ may be defined as follows:

$$DB(K) = \frac{1}{K} \sum_{k=1}^{K} R_k \quad \text{for} \quad K \in \mathbb{N} \tag{14}$$

where

$$R_k = \max_{j=1,\ldots,K, j\neq k} \left(\frac{S_k + S_j}{d_{k,j}} \right) \quad \text{for} \quad k \in [1, \ldots, K] \tag{15}$$

and

$$S_k = \frac{1}{\sum_{i=1}^{N} w_{k,i}} \sum_{i=1}^{N} w_{k,i} \parallel x_i - \bar{x}_k \parallel \quad \text{for} \quad k \in [1, \ldots, K] \tag{16}$$

as well as

$$d_{k,j} = \parallel \bar{x}_k - \bar{x}_j \parallel \tag{17}$$

For each cluster C_k an utmost similar cluster—regarding their intra-cluster error sum of squares—is searched, leading to R_k. The index then defines the average over these values. In contrast to the aforementioned cluster indexes, here, the minimal observed index indicates the best cluster solution.

Krzanowski-Lai

Krzanowski and Lai [6] developed a cluster index that, similar to the index of Calinski and Harabasz [2], is based on the squared differences of all objects in a cluster from their respective cluster center—*trace* $\mathbf{W}$. The authors define $DIFF(K)$ as the difference between a clustering of the data in K and a clustering in $K-1$ clusters. Let J be the number of variables that has been measured on each $\mathbf{x_i} \in \mathbf{X}$ and *trace* $\mathbf{W}_K$ the sum of squares function that corresponds to the clustering in K clusters, their measure $DIFF(K)$ is then defined as follows:

$$DIFF(K) = (K-1)^{\frac{2}{J}} \cdot trace\ \mathbf{W}_{K-1} - K^{\frac{2}{J}} \cdot trace\ \mathbf{W}_K \tag{18}$$

Here, the introduction of the normalizing factor $\frac{2}{J}$ is derived from the observation that—given independently uniformly distributed measurements on each variable $j \in [1, \ldots, J]$—the optimal clustering of the data will reduce the sum of squares exactly by this factor [6, p.25].

The authors claim that if there exists an optimal clustering solution in K^* groups, the value of $DIFF(K^*)$ should be comparably large and positive (see index of Calinski and Harabasz for further explanation). In contrast, all values of $DIFF(K)$ for $K > K^*$ will have rather small values (maybe even negative), while values for $K < K^*$ will be rather large and positive. Bringing these observations together the index $KL(K)$ is defined as follows:

$$KL(K) = \left| \frac{DIFF(K)}{DIFF(K+1)} \right| \tag{19}$$

The optimal cluster solution is then indicated by the highest value of $KL(K)$.

Figure of Merit

Coming from a gene expression background, the Figure of Merit [7] is based on the assumption that the validity of a cluster is certainly increasing in value if in a second experiment the same genes would group together and reveal a similar pattern of expression. Following a bootstrapping or jackknife approach, one may assume that a cluster algorithm is successively applied on a set of genes whereby in each iteration one experimental condition—in exact terms a feature of each classification object, or a column of the data matrix—is left out. If a cluster algorithm would have assigned each object to a cluster just by chance, it seems logical that omitting a condition will lead to different results. Otherwise, it is likely that two cluster results reveal a similar structure if the dependence on the left-out feature is small.

Let in the following $\mathbf{X} = \{\mathbf{x_i}, i = 1, \ldots, N\}$ denote a set of N classification objects, each having the dimension $P \in \mathbb{N}$, such that $x_{i,j}$ is the j-th feature of $\mathbf{x}_i$, $j \in 1, \ldots, P$; furthermore, let there be a number of clusters $K \in \mathbb{N}$ whereby $\mathbf{W}(\mathbf{X}) = [w_{k,i}]_{K \times N}$ describes the clustering of

the data. Assuming that a clustering has been performed with a data matrix where the j-th feature has been omitted, the Figure of Merit is defined as follows:

$$FOM(j,K) = \sqrt{\frac{1}{N}\sum_{k=1}^{K}\sum_{i=1}^{N} w_{k,i}\left(x_{i,j} - \overline{C_{k,j}}\right)^2} \tag{20}$$

where

$$\overline{C_{k,j}} = \frac{1}{N}\sum_{i=1}^{N} w_{k,i}x_{i,j} \tag{21}$$

denotes the arithmetic mean in feature j of all objects of cluster k.

To avoid a bias towards the overall number of clusters, the so called “adjusted Figure of Merit” takes this amount K into account:

$$\text{adjusted } FOM(j,K) \cdot \frac{1}{\sqrt{\frac{N-K}{N}}} \tag{22}$$

If the calculation is iterated over all P features of the classification objects, the “aggregate Figure of Merit” can be computed:

$$\text{aggregate } FOM(K) = \sum_{j=1} PFOM(j,K) \tag{23}$$

The authors state that in the outcome “A small figure of merit indicates a clustering algorithm having high predictive power. We compare clustering algorithms with the same number of clusters, and over a range of number of clusters” [7, p.310].

References

[1] Hastie T, Tibshirani R, Friedman J: *The Elements of Statistical Learning.* Springer Series in Statistics, Springer 2001.

[2] Calinski RB, Harabasz J: **A dendrite method for cluster analysis**. *Communications in Statistics* 1974, **3**:1–27.

[3] Maulik U, Bandyopadhyay S: **Performance Evaluation of Some Clustering Algorithms and Validity Indices**. *IEEE T. Pattern. Anal.* 2002, **24**(12):1650–1654.

[4] Davies DL, Bouldin DW: **A cluster separation measure**. *IEEE T. Pattern. Anal.* 1979, **1**:224–227.

[5] Halkidi M, Batistakis Y, Vazirgiannis M: **Cluster Validity Methods: Part I & II**. *SIGMOD Rec.* 2002, **31**(2):40–45.

[6] Krzanowski WJ, Lai YT: **A Criterion for Determining the Number of Groups in a Data Set Using Sum-of-Squares Clustering**. *Biometrics* 1988, **44**:23–34.

[7] Yeung K, Haynor D, Ruzzo W: **Validating clustering for gene expression data**. *Bioinformatics* 2001, **17**:309–318.
