# Supplementary material for: A guide through the computational analysis of isotope-labeled mass spectrometry-based quantitative proteomics data: an application study
Source: Proteome Sci. 2011 Jun 11;9:30. doi: 10.1186/1477-5956-9-30 (PMC3142201; doi:10.1186/1477-5956-9-30)
Supplement: Additional file 8 — A walk through QuPE. A walk-through guide introducing some of the functionality of the rich internet application QuPE. [file 1477-5956-9-30-S8.pdf]

http://qupe.cebitec.uni-bielefeld.de

QuPE

# A walk through QuPE

## A guide through the computational analysis of isotope-labeled mass spectrometry-based quantitative proteomics data

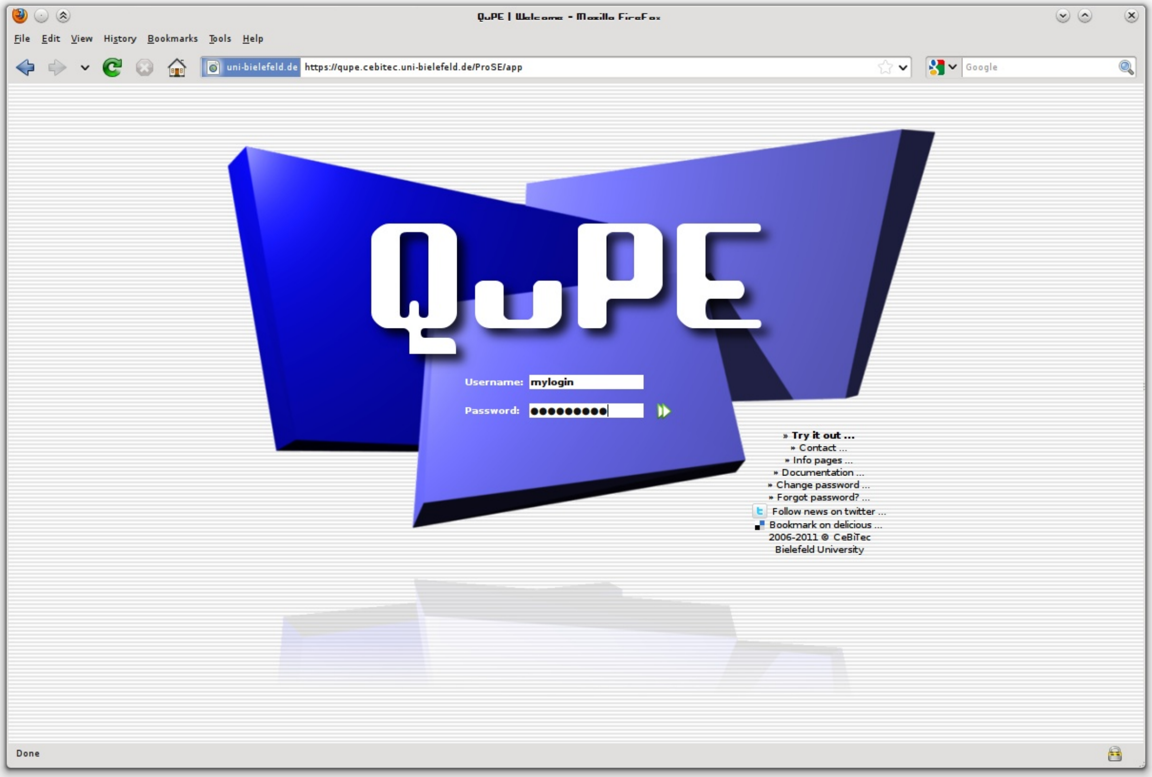


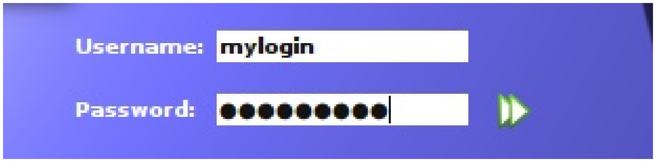


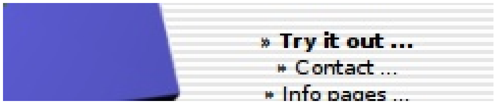


In the following, we would like to introduce you to some of the functionality of QuPE - a rich internet application to store and analyze quantitative proteomics experiments. This walkthrough aims to guide through the system and demonstrates how to browse analysis results. QuPE is best displayed using Mozilla Firefox, but other browsers such as Google Chrome or Internet Explorer can, of course, also be used.

To **log into** the system, please first enter your **username** and **password** or click on the "**Try it out...**" link on the right side of the logo.

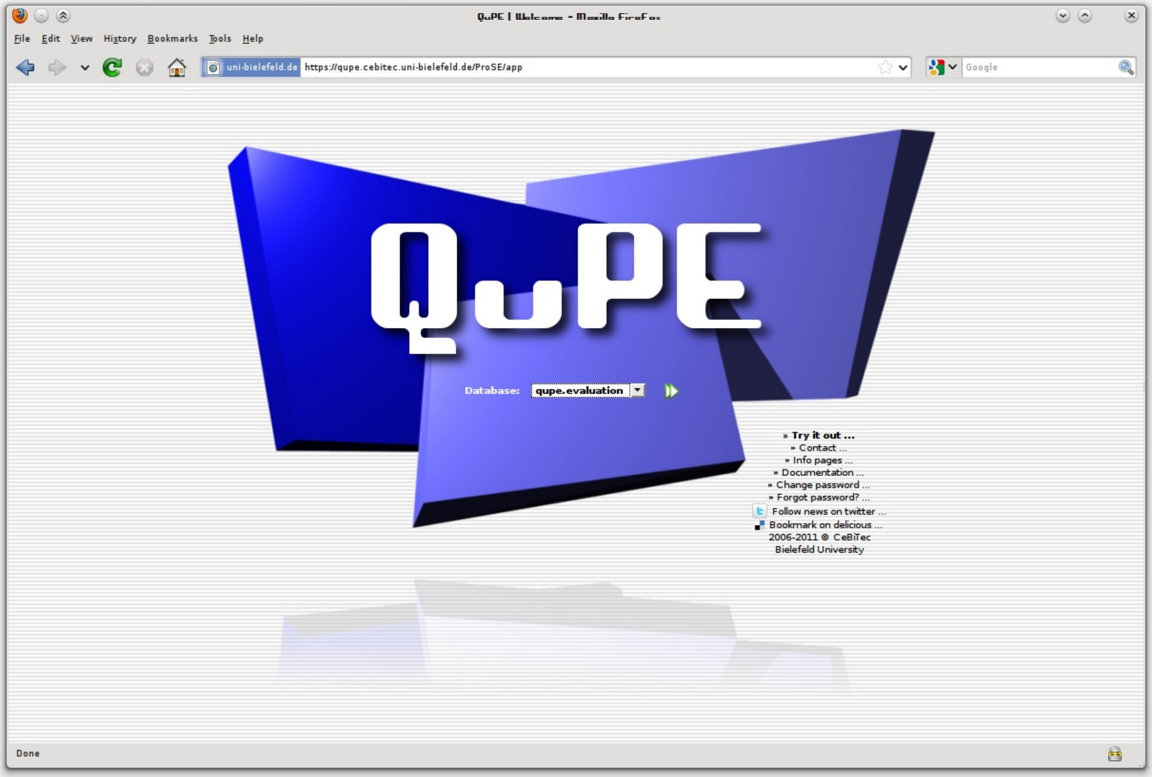


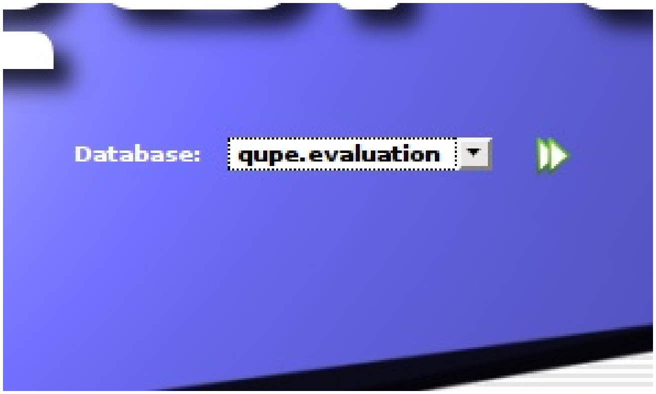


After log-in please choose a database. Please select for example "qupe.evaluation", to view the analysis results presented in "A guide through the computational analysis of isotope-labeled mass spectrometry-based quantitative proteomics data: an application study".

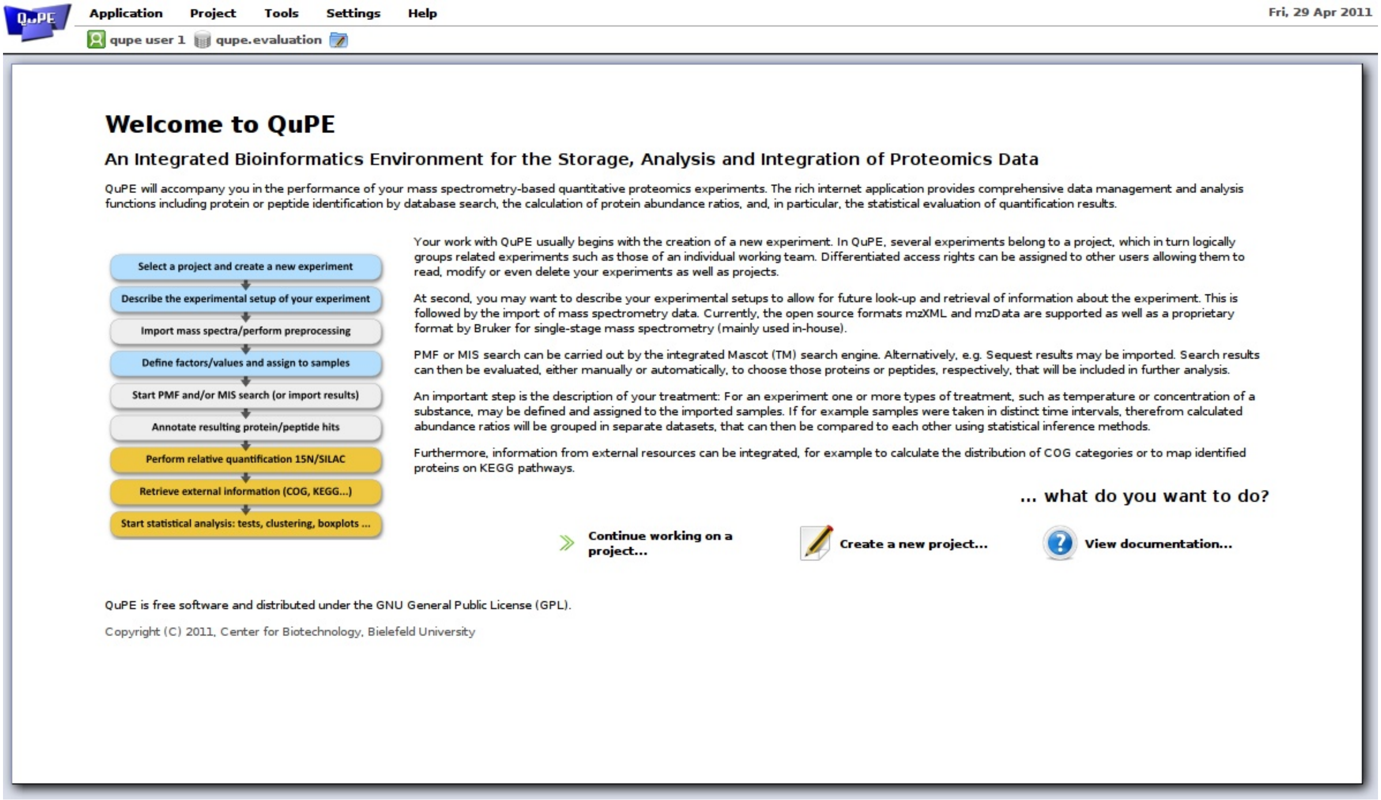


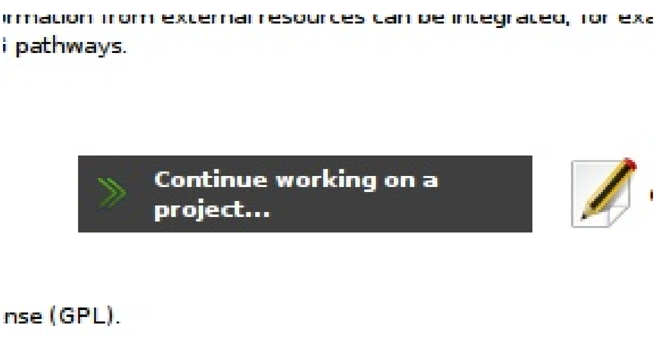


Welcome to QuPE! The software helps to organize and manage your quantitative proteomics experiments: In QuPE, several experiments belong to a project, which in turn logically groups related experiments such as those of an individual working team. Differentiated access rights can be assigned to other users allowing them to read, modify or even delete your experiments as well as projects.

To proceed through the guide, please select "Continue working on a project...".

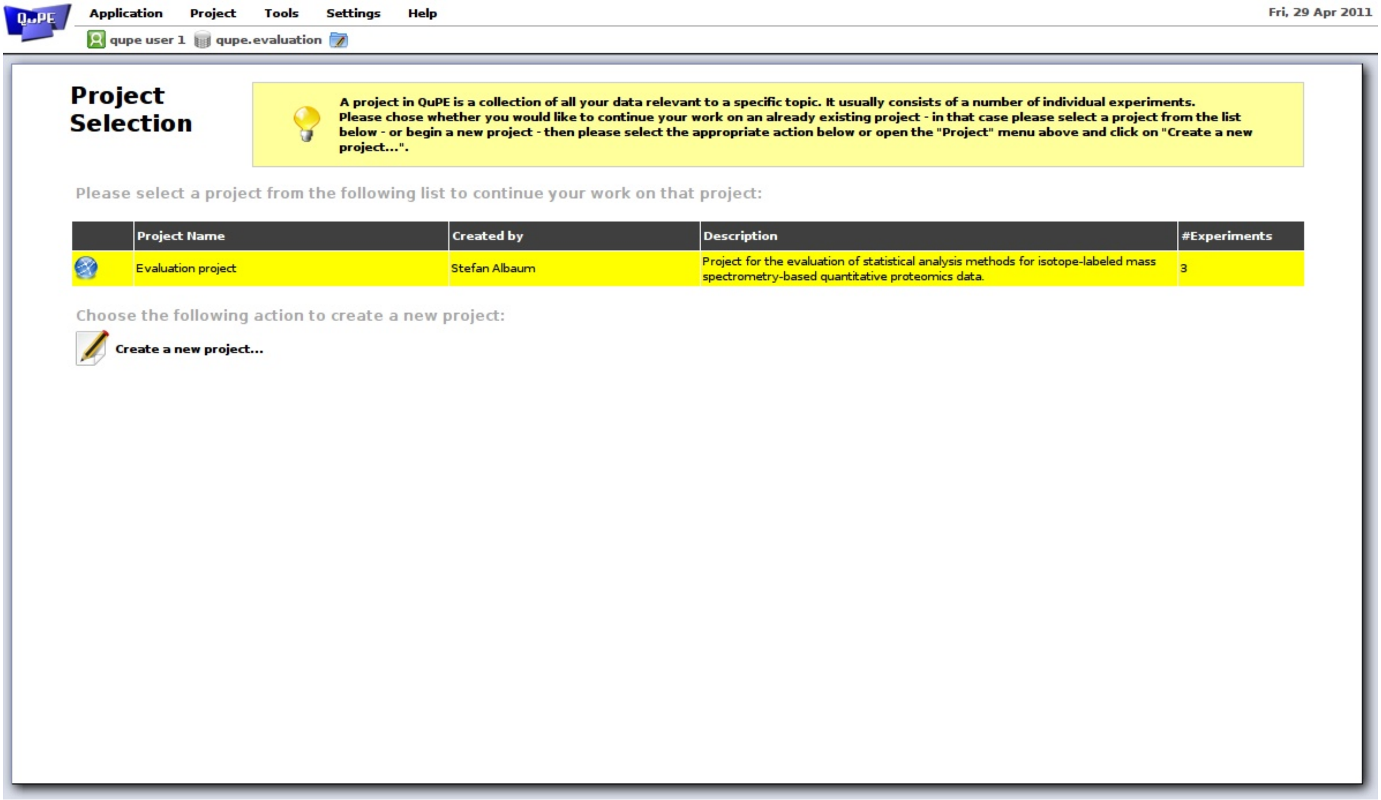


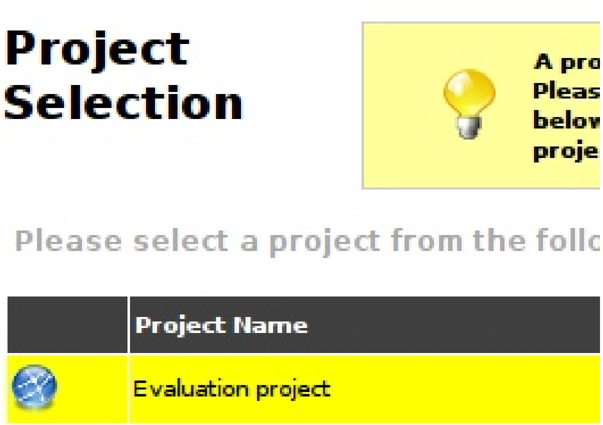


The following page lists all projects within the selected database - given, of course, that you have the appropriate access rights. Usually, you will only want to create a new personal or workgroup-related project in the beginning of your work with QuPE. Afterwards, you probably only want to create new experiments within this project.

To continue your work on an existing project, just click on the projects entry in the list, in our case the yellow-highlighted "Evaluation project".

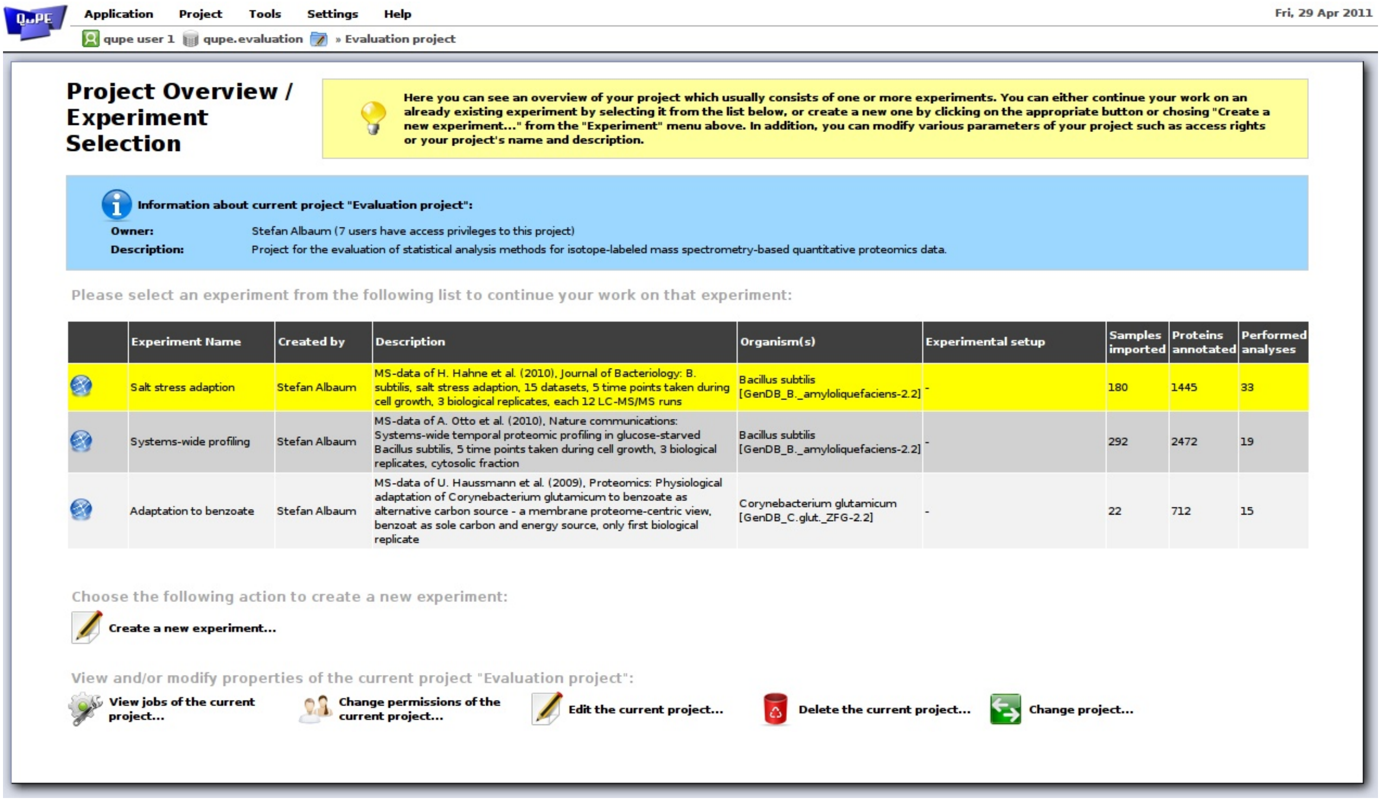


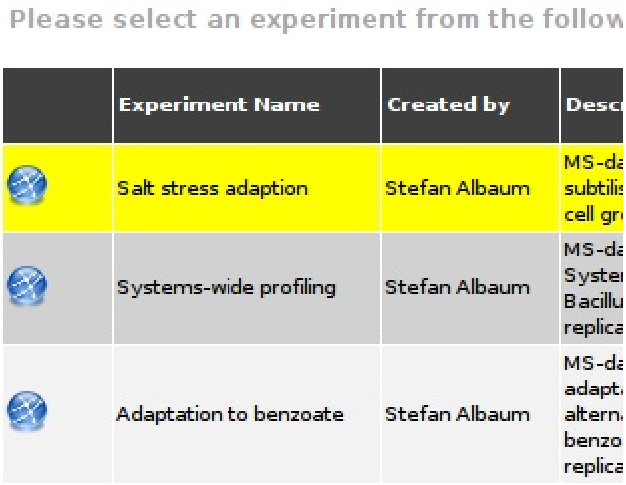


After selection of a project, again a list - here of all experiments belonging to the selected project - is displayed. In this case, three experiments are shown which correspond to the three experiments of the refered manuscript: experiment A is named "Salt stress adaption", experiment B "Systems-wide profiling", and experiment C "Adaption to benzoate". Choose one of the experiments, e.g. the first, to browse its results.

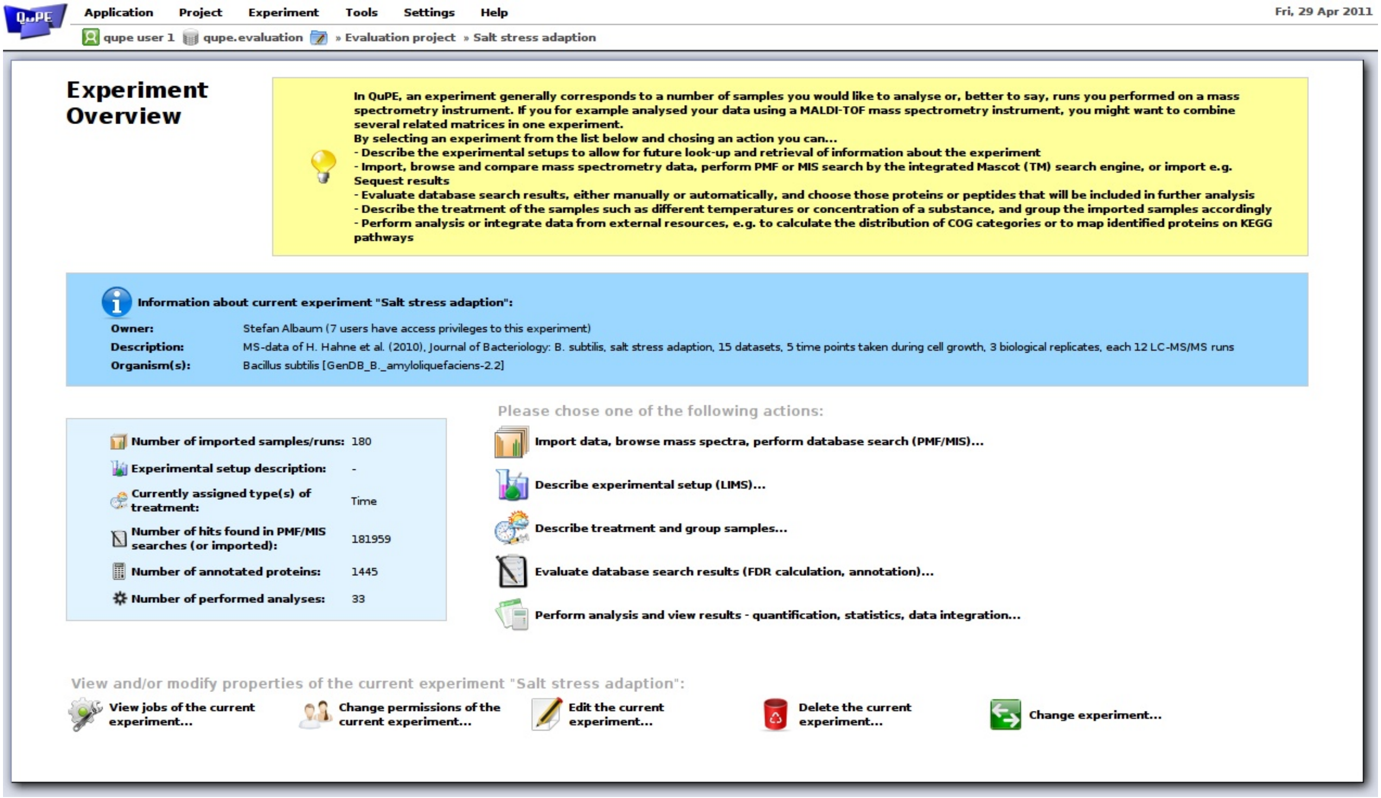


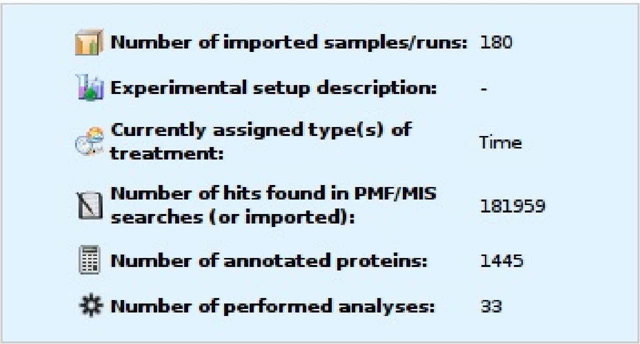


An experiment generally corresponds to a number of samples you would like to analyse or, better to say, runs you performed on a mass spectrometry instrument. If you for example analysed your data using a MALDI-TOF mass spectrometry instrument, you might want to combine several related matrices in one experiment, or, in case of LC-MS/MS, several runs.

The blue information box on the left side displays information about the current number of imported samples, observed database hits, and performed analysis.

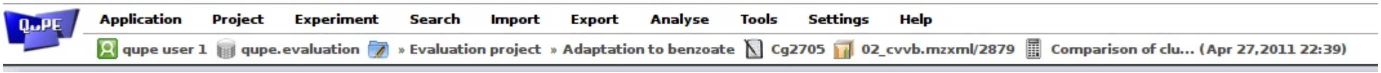


**Basic navigation in QuPE**

In general, all navigation in QuPE can be done using the menu in the title bar:

- Use the Application menu to change the database and to log out
- The project as well as the experiment menu allow to change your current project or experiment, respectively, but also the creation of a new one
- Other menus provide options for data import, export, and allow to start analyses
- Have a look at the help menu to browse related resources and more documentation (please allow **pop-up windows** therefore)

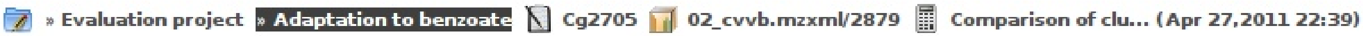


The status bar allows to quickly navigate to the project or experiment overview page by clicking on the appropriate entry. If a specific spectrum (02_cvvb.mzxml/2879 in our example) or protein (e. g. Cg2705) has been selected somewhere, a click will directly provide additional information.

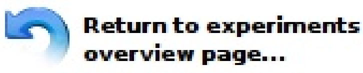


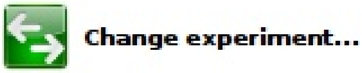


Two very important actions:
Select the "Return to experiments overview page..." to get back to the overview and browse to another page, and "Change experiment..." to switch to another experiment.

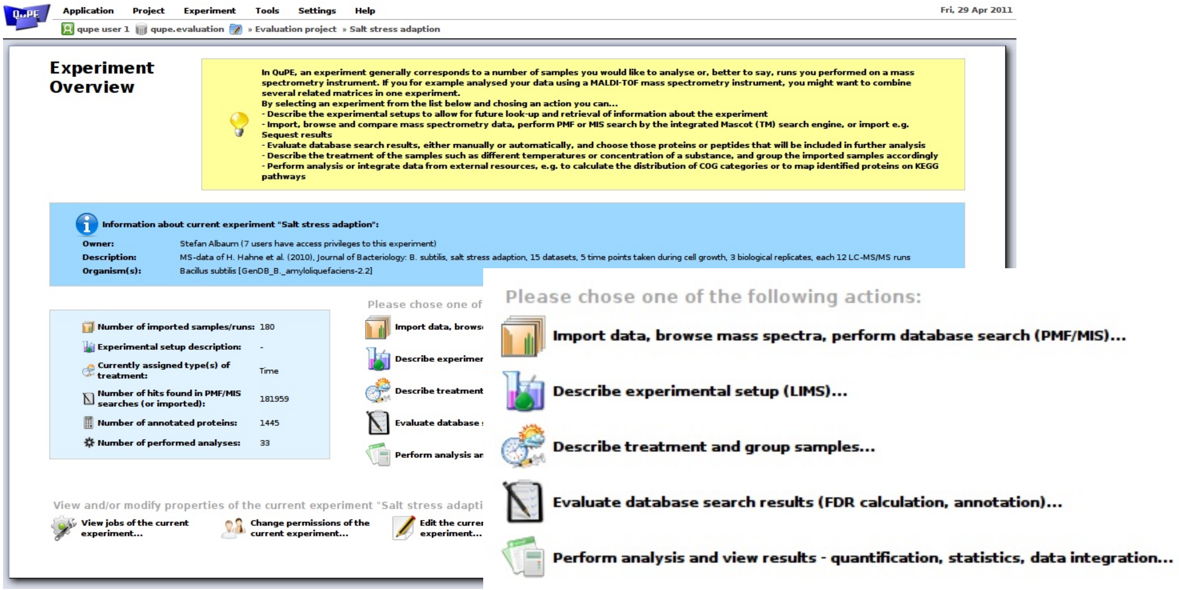


Basically, the workflow of an experiment in QuPE consists of five steps:

1. Import of mass spectrometry data followed by a PMF or MIS search using the integrated Mascot (TM) search engine or import e.g. of Sequest results
2. Description of the experimental setups to allow for future look-up and retrieval of information about the experiment
3. Definition of factors and values to organize samples for further analysis steps. An example for a factor would be "time" with levels 1, 2, and 3h
4. Evaluation of search results, either manually or automatically to choose a set of proteins or peptides, respectively, that will be included in further analysis
5. Calculation of abundance ratios, statistical analysis, integration of annotation data from external resources, e.g. to calculate the distribution of COG categories or to map identified proteins on KEGG pathways

**Please note:** the following three pages explain how to browse mass spectra, to group samples, and to evaluate database search results (PMF/MIS). You may want to skip over these pages and directly proceed with the walk through the analysis results.

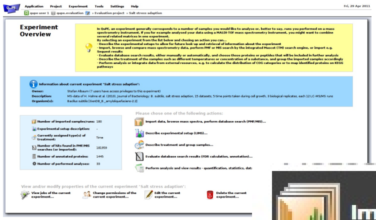

On the experiment overview page, select the action "Import data, browse mass spectra, perform database search..." to browse imported mass spectra...

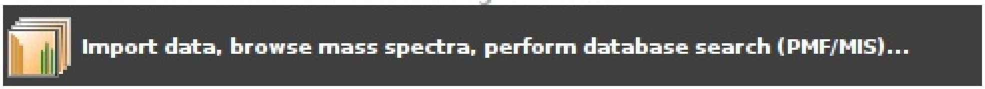


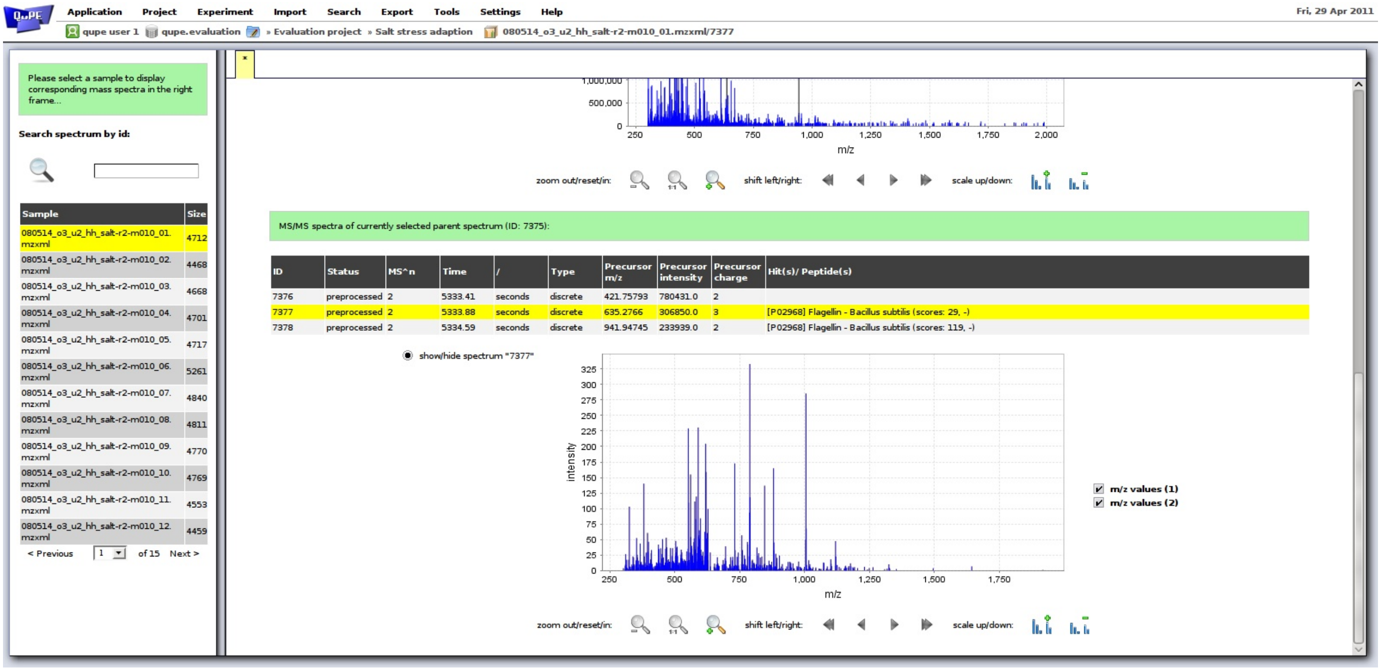


Working with mass spectra: In the left sidebar all imported samples (e. g. LC runs) are displayed, select one to browse the corresponding mass spectra. In general, for each imported file (mzData, mzXML,..) a separate sample has been created. On the right side all spectra belonging to the selected sample will be displayed. This includes MS level one spectra, and - if applicable - the child spectra (MS2, SIM, Zoom, etc.) associated to the selected MS1 scan. Buttons below the depicted peak lists provide zoom functionality. Checkboxes beside the spectrum allow to partly hide peak lists, for example to select only the precursor positions.

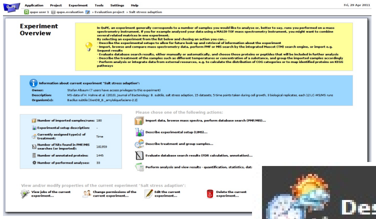

Any analysis demands grouping of samples which have been measured under the same conditions. Therefore, please choose the action "Describe treatment and group samples..." on the experiments overview page.

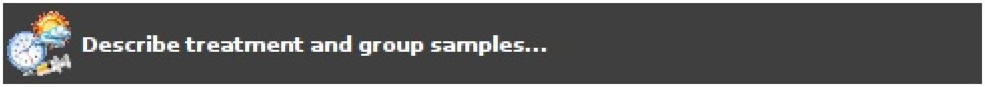


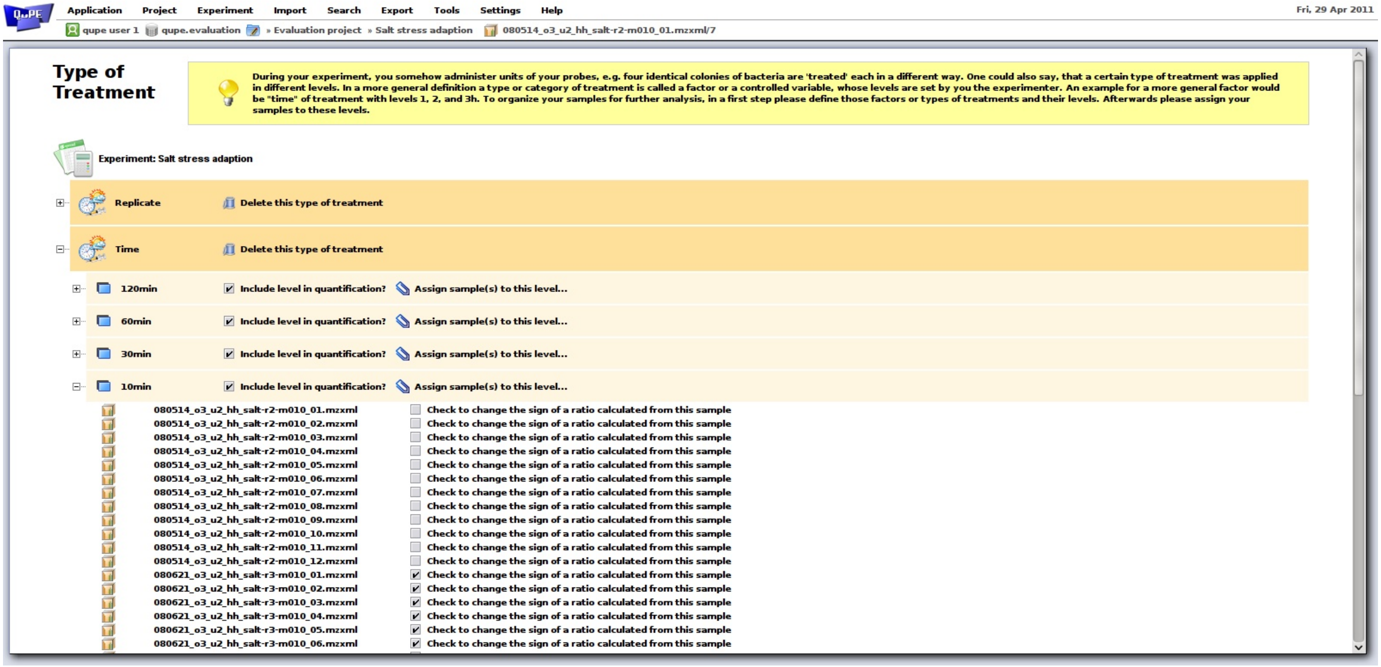


Before any analysis such as a statistical test can be performed, QuPE needs to know which samples have been measured under the same conditions. This is, for example, a distinct time point, a strain - e. g. mutant or wildtype -, or a specific growth medium. On this page, the model or basis of the experiment is described and set up. This will then be used in any further analysis, i. e. if abundance ratios are calculated these will be grouped into datasets according to the here described model, an analysis of variance will refer to this model, as well as a cluster analysis that will group the input data accordingly.

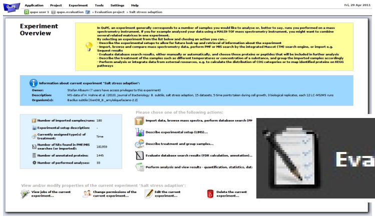

QuPE allows the import of search results, e. g. in form of a DTASelect-filter file, and has an integrated Mascot search engine to perform Peptide Mass Fingerprinting  and MS/MS ion search.

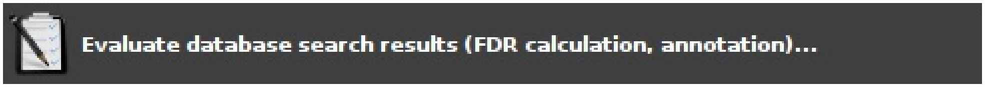


Click on "Evaluate database search results..." to browse and work on all observed database hits.

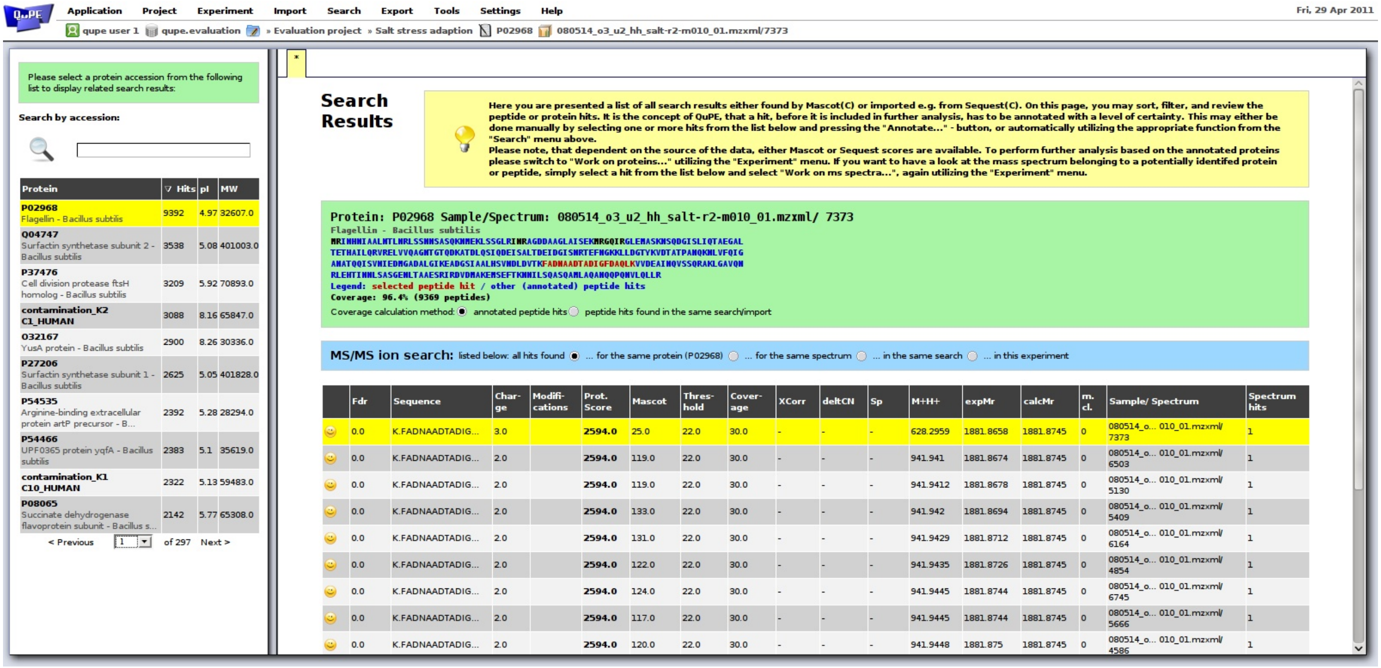


The list on the left side displays all proteins found in database searches. Click on a table header to sort the list, e.g. by the number of observed hits. Select a protein from the list to show all related database hits (protein hits in case of PMF, peptide hits in case of MIS) on the right side of the page. Again you may click on the table headers to sort the list. Above the list of all protein/peptide hits options are provided for filtering. Instead of having a look at all hits found for the currently selected protein you may thereby investigate whether other hits have been found for the same spectrum. Additional information is displayed when a hit was selected: If appropriate accession numbers are found, links to uniprot or ncbi will be available, and in case search results were obtained by a Mascot search, links to these search results will be shown.

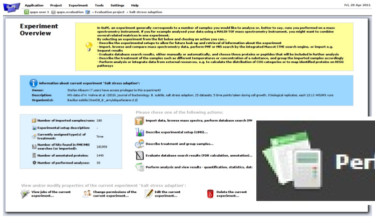

Please click on "Perform analysis and view results..." on the experiments overview page to start quantification of your proteins, conduct a statistical test or a hierarchical clustering.

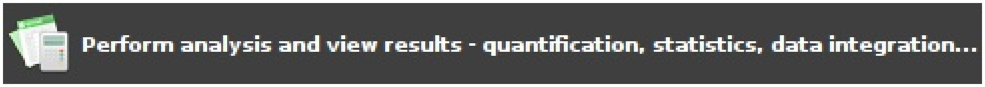


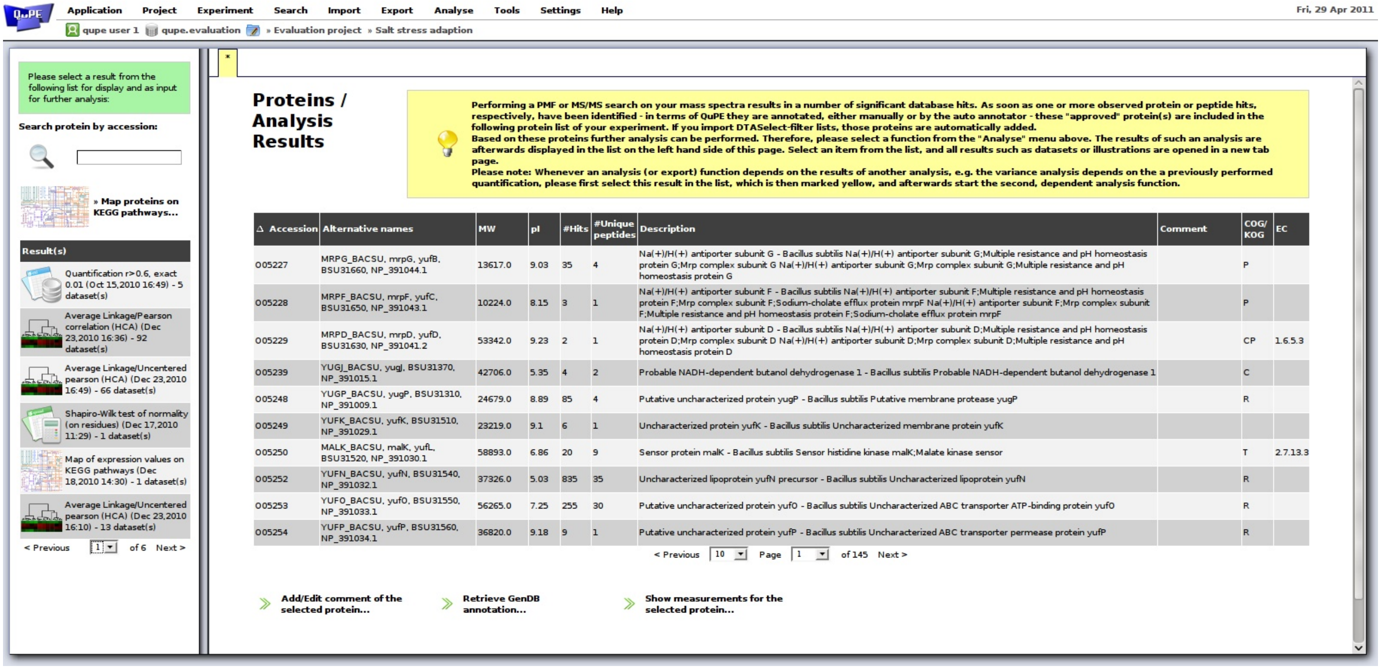


After mass spectra have been imported in QuPE, a database search has been conducted (or protein identifications have been imported), resulting hits have been processed (e. g. filtered using an FDR threshold), and the model of the experiment has been described (i. e. samples recorded under the same condition have been grouped together), next steps are quantification, data integration and analysis. Found on this page is, firstly, a table showing all identified ('annotated') proteins. Any further analysis will be based on this list of proteins. Secondly, different kinds of analysis can be started and the results e. g. of a protein quantification, a statistical test or a hierarchical clustering can be viewed. In our example, the results for experiment A "Salt stress adaption" are shown. To begin with, a tool has been invoked to enrich our knowledge of the identified proteins with information from "other" databases like uniprot, ncbi and our gene annotation system GenDB.

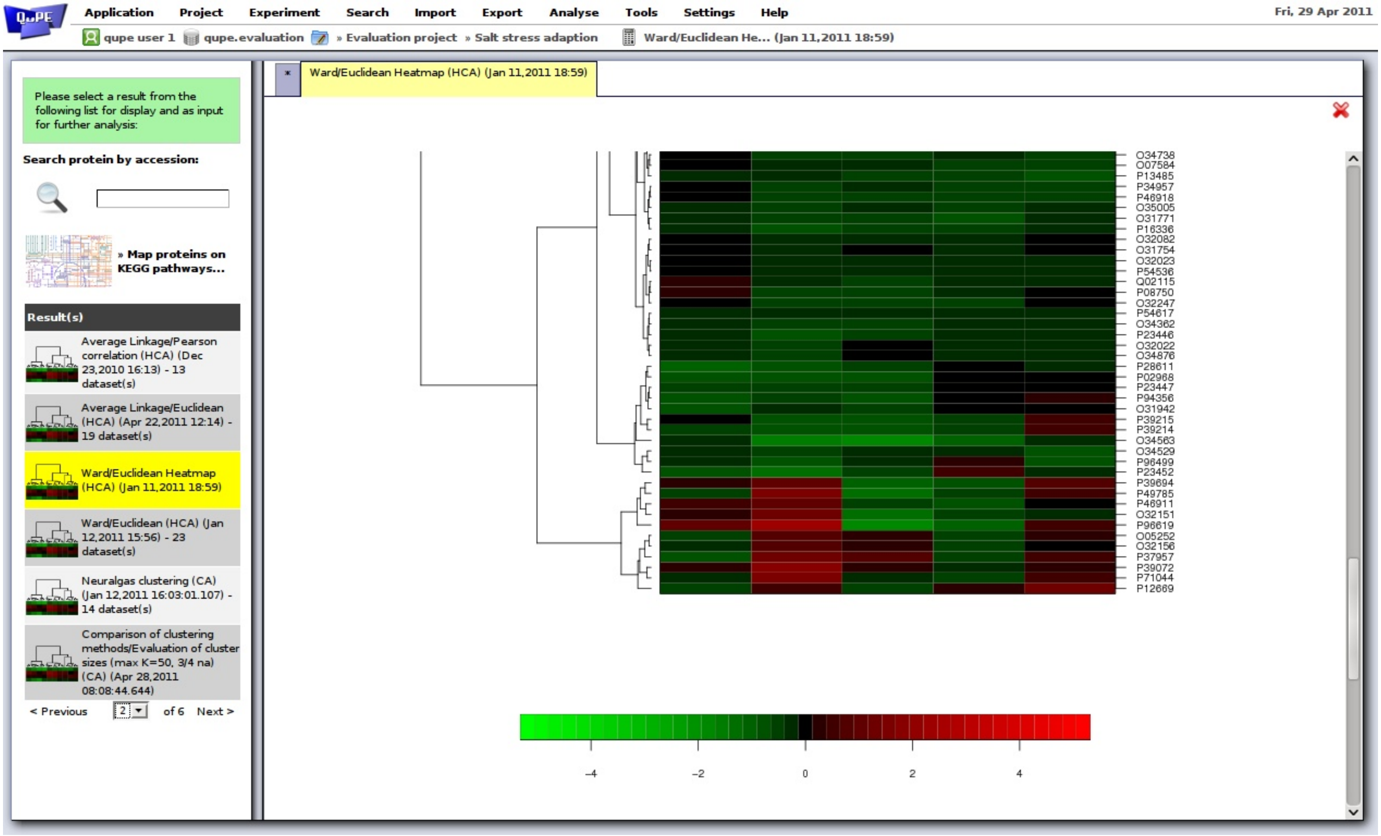


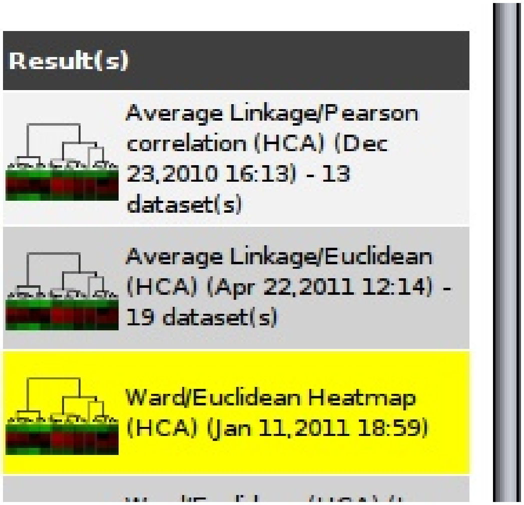


If a result has been selected the main window on the right side utilizes tabs to display the selected results object and other information. In our example, the heatmap resulting from a hierarchical cluster analysis using Ward and Euclidean distances is displayed. Click on the tab named "*" to switch back to the protein overview. To browse all results of an experiment, use the table navigation and switch to the "previous" or the "next" page of results. In the beginning always the latest analysis results on the last page are displayed.

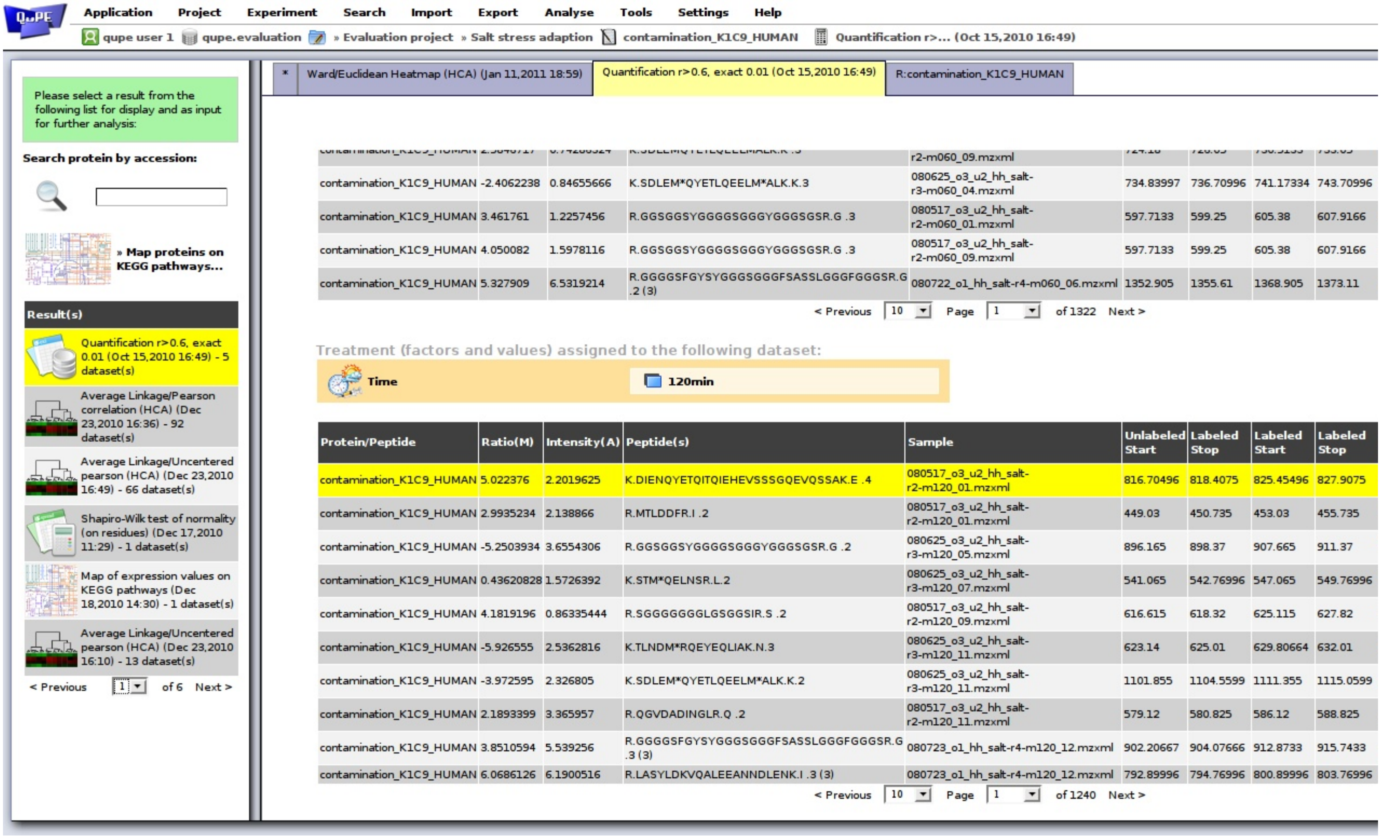


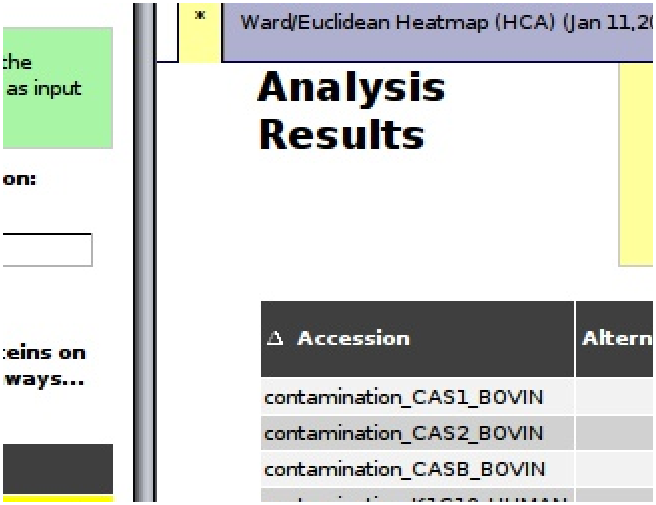


Here, a quantification result has been selected. If a protein is chosen from the list, this protein is immediately propagated to other "listening" parts of the application, such as the protein overview page, where the corresponding protein will be highlighted. (Even if the view is changed, for example switched back to the search results or ms spectra view, the corresponding scan or hit will automatically be highlighted).

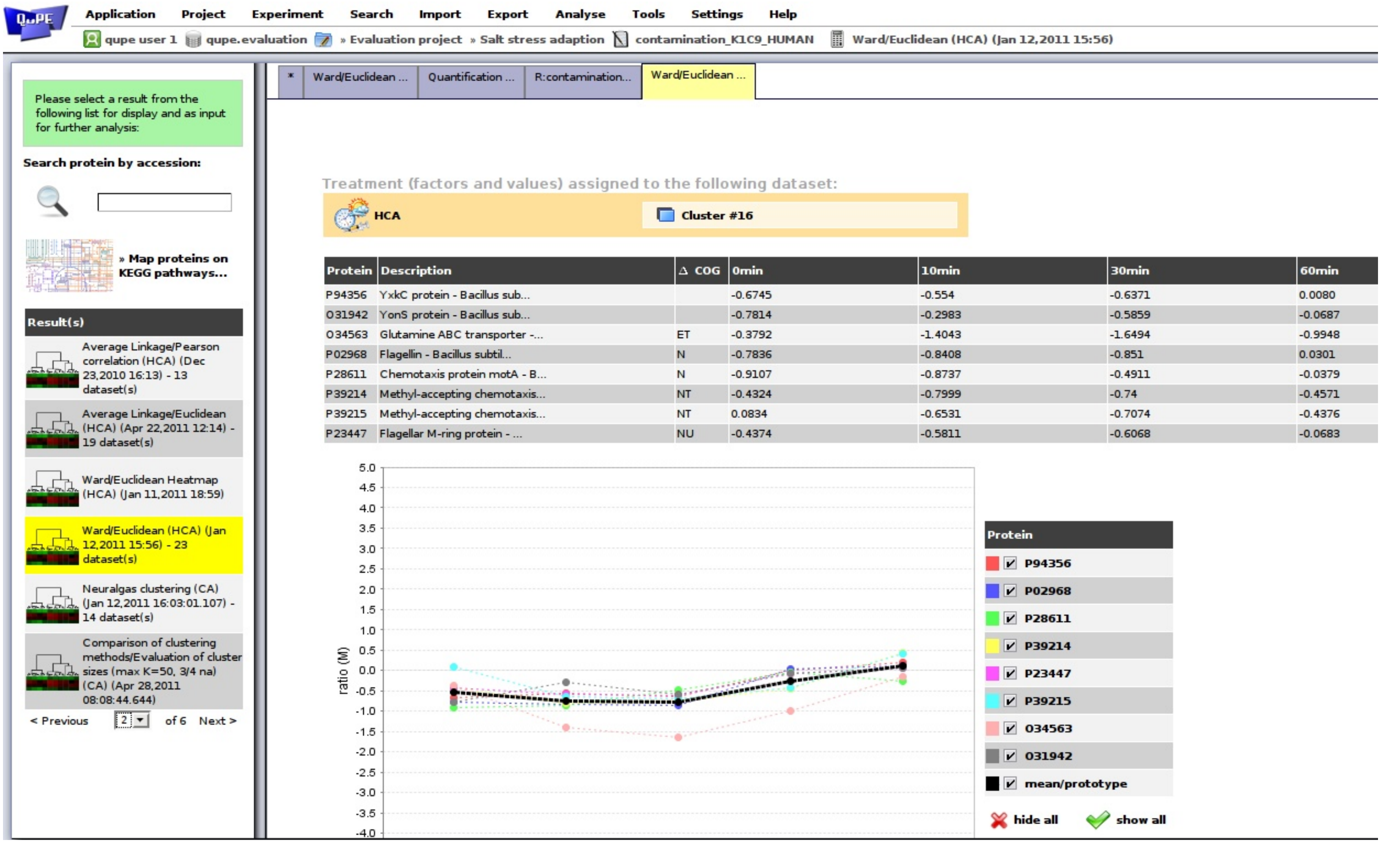


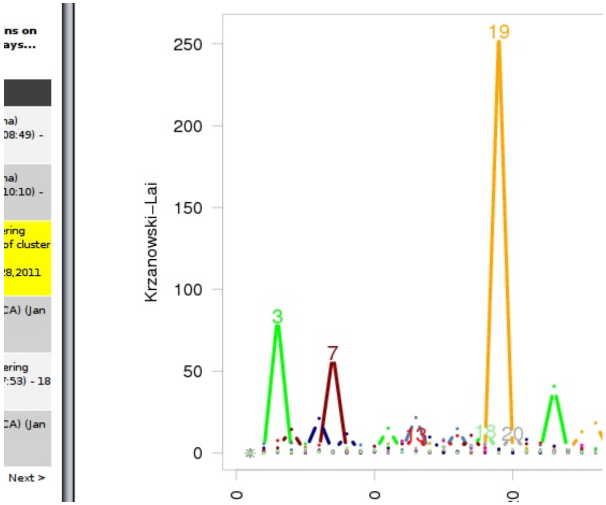


Here, the results of a hierarchical cluster analysis using Ward and Euclidean distances are shown. The resulting cluster tree has been 'cut' in 23 clusters. This was indicated as an optimal clustering by the cluster index of Krzanowski and Lai.

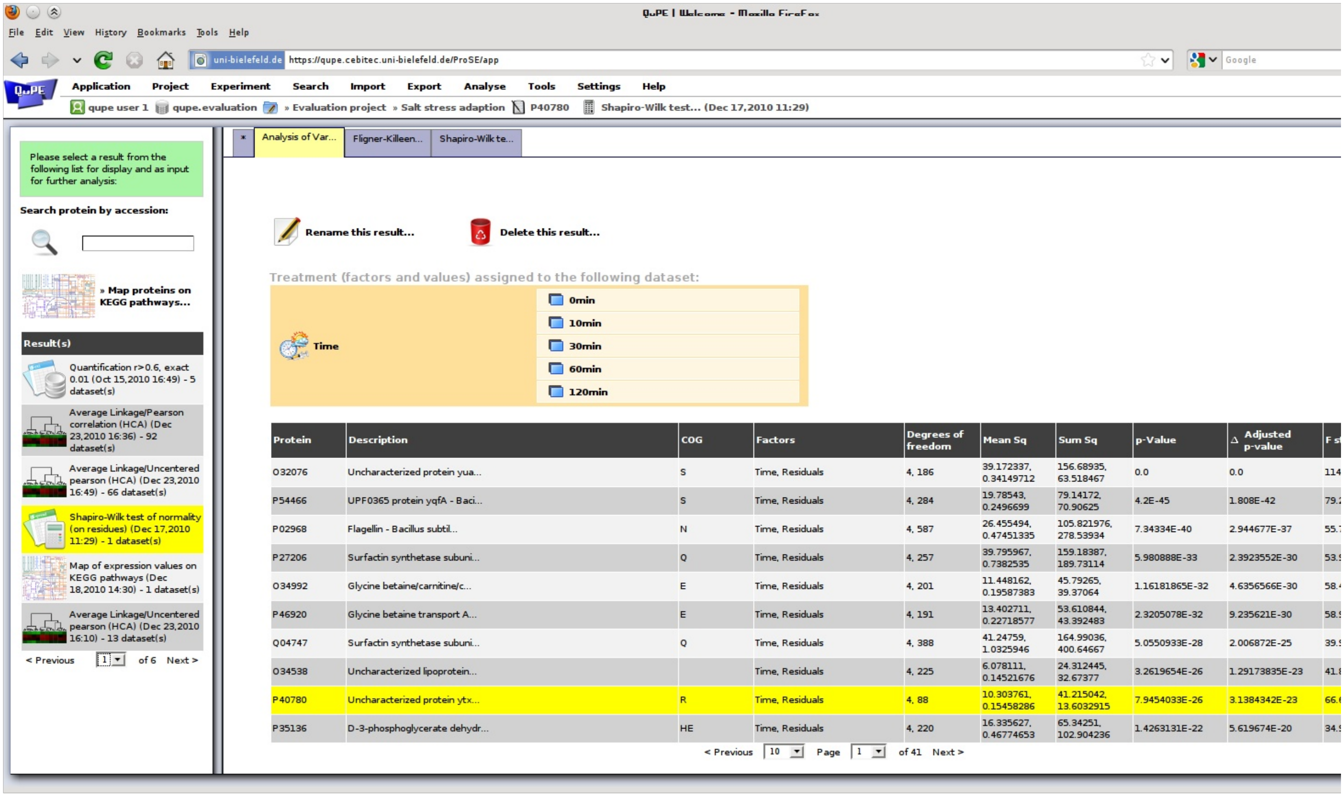


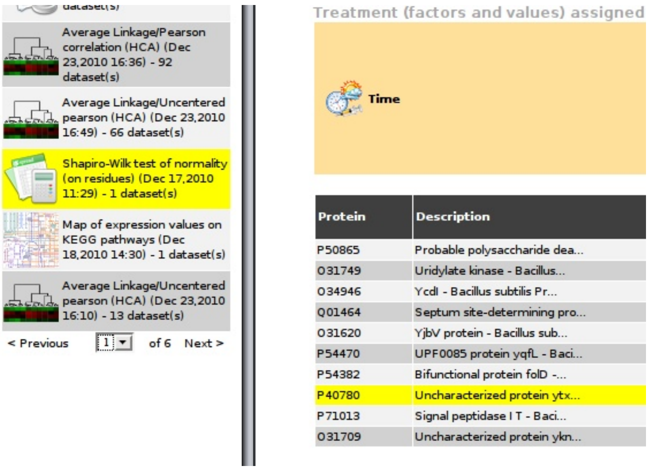


This page shows the results of an ANOVA sorted by an increasing adjusted p-value. Again, if a protein is selected in the tables, the same will be highlighted for example in a further open tab which shows the results of a Fligner-Killeen test of homogeneity of variances.

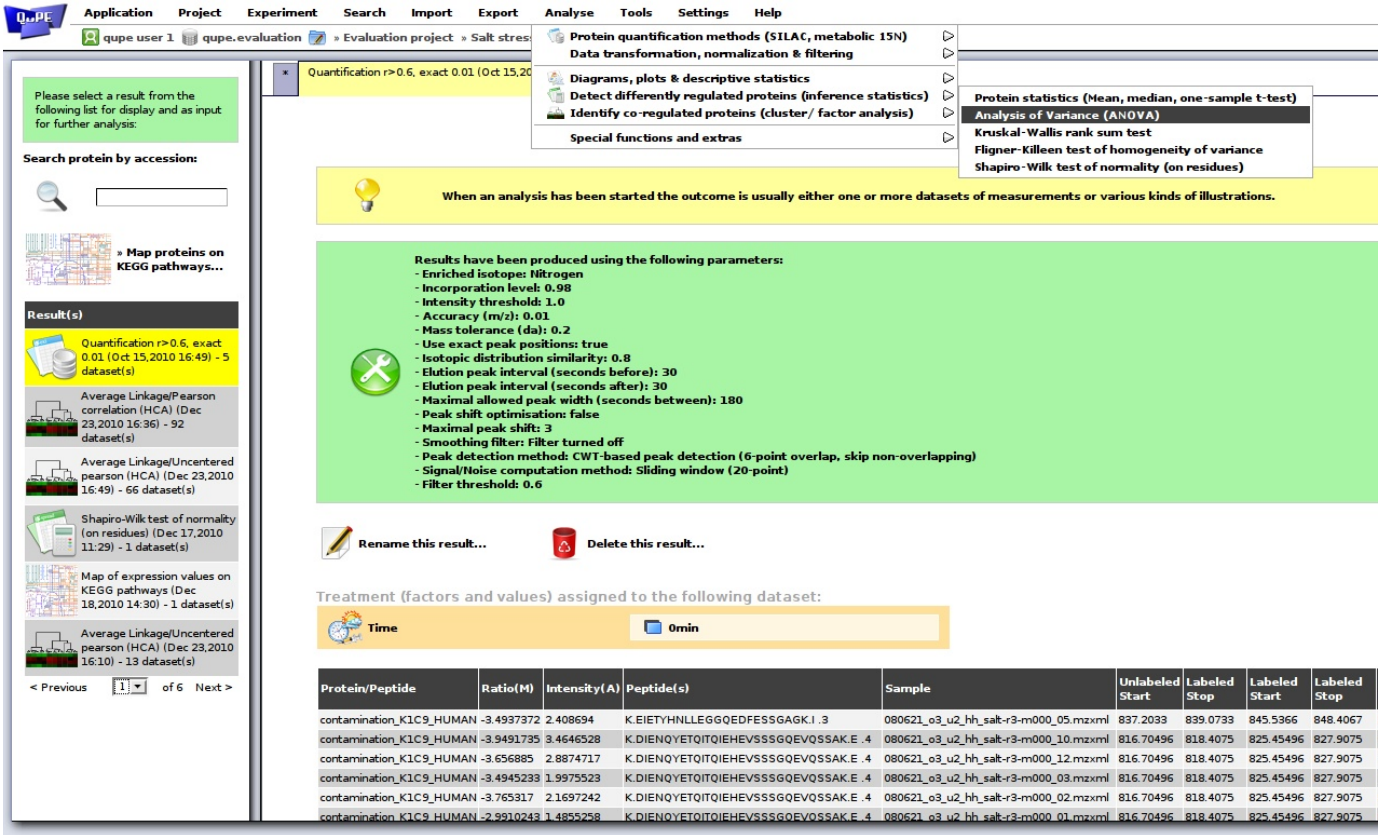


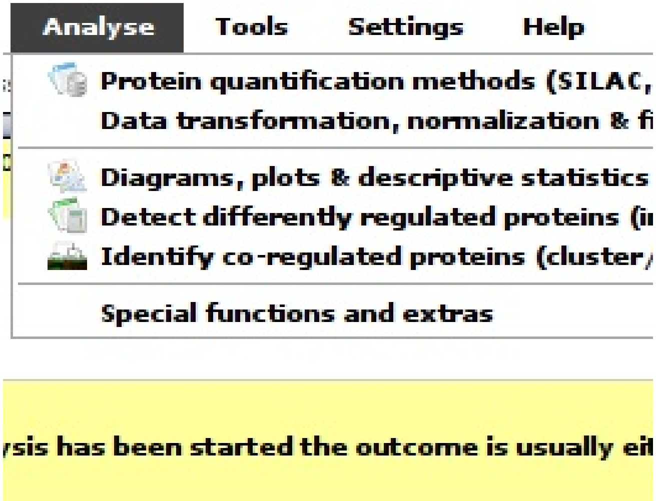


To perform a new analysis, e. g. an Analysis of Variance, simply select the appropriate function from the "Analyse" menu. This is, of course, only possible if you have write access to the current experiment.

Further information on how to set up a new experiment, import data, perform MIS/PMF database searches or to conduct analyses can be found online in the QuPE documentation (e. g. select the "Documentation" entry from the "Help" menu).

# Thank you very much for your interest in QuPE

QuPE@CeBiTec.Uni-Bielefeld.de
If you have further questions please do not hesitate to contact us.
